# Precision immunomodulation of viral myocarditis: a spatiotemporal intervention paradigm based on smart biomaterials

**DOI:** 10.3389/fimmu.2026.1939896

**Published:** 2026-09-08

**Authors:** Ying Dai, Bo Han

**Affiliations:** Department of Pediatric Cardiology, Shandong Provincial Hospital Affiliated to Shandong First Medical University, Jinan, Shandong, China

**Keywords:** immunometabolism, precision immune regulation, regulated cell death, smart biomaterials, viral myocarditis

## Abstract

Viral myocarditis (VMC) represents a primary etiology of sudden cardiac death (SCD) in young populations and significantly contributes to the development of dilated cardiomyopathy (DCM). The clinical prognosis of VMC is contingent upon the precise orchestration of pathogen clearance and the prevention of deleterious immune activation. Disease progression is characterized by multifaceted immunometabolic reprogramming, regulated cell death (RCD) pathways (including pyroptosis and ferroptosis), and complex crosstalk between damaged cardiomyocytes and infiltrating immune cells. However, conventional broad-spectrum immunosuppressants often fail to provide substantial clinical benefits due to a lack of spatiotemporal specificity, which complicates the delicate balance between anti-inflammatory efficacy and the preservation of antiviral immunity. This review delineates the paradigm shift in VMC therapeutic strategies, transitioning from systemic pharmacotherapy toward precision immunomodulation mediated by advanced biomaterials, such as functionalized cardiac patches, engineered exosomes, and piezoelectric materials. By integrating breakthroughs in immunology and bioengineering, these emerging platforms offer a promising avenue for targeted intervention during the early stages of VMC. Such approaches aim to suppress pathological inflammation without compromising intrinsic antiviral capacity, ultimately preventing the progression from acute VMC to irreversible heart failure.

## 1 Introduction

Viral myocarditis (VMC) represents a formidable challenge in clinical cardiovascular medicine and remains a leading etiology of SCD among adolescents and young adults (1). The clinical spectrum of VMC is remarkably heterogeneous, ranging from subclinical, asymptomatic electrocardiographic (ECG) changes to fulminant heart failure (2). While many patients achieve complete resolution following the acute phase, a substantial subset—underpinned by persistent subclinical inflammation—gradually progresses to dilated cardiomyopathy (DCM). As an end-stage cardiac condition, DCM is characterized by ventricular dilatation and progressive contractile dysfunction, which may ultimately necessitate cardiac transplantation in severe cases (3). Despite substantial advancements in heart failure management, targeted therapeutic strategies addressing the underlying immunopathological mechanisms of VMC remain elusive. Consequently, identifying effective interventions to halt the progression from acute inflammatory injury to chronic, irreversible heart failure represents a critical unmet clinical need (4). The abbreviations used in this article are summarized in **Table 1**.

**Table 1:** List of abbreviations.

Full name	Abbreviation	Full name	Abbreviation
Viral myocarditis	VMC	Antibody-dependent cellular cytotoxicity	ADCC
Dilated cardiomyopathy	DCM	Interferon-β	IFN-β
Sudden cardiac death	SCD	Interferon-stimulated genes	ISGs
Electrocardiographic	ECG	Interleukin-1β	IL-1β
Damage-associated molecular patterns	DAMPs	Canakinumab Anti-inflammatory Thrombosis Outcomes Study	CANTOS
Toll-like receptor 3	TLR3	Interleukin-6	IL-6
Double-stranded RNA	dsRNA	Intravenous immunoglobulin	IVIG
Interferons	IFNs	Good Manufacturing Practice	GMP
Pattern recognition receptors	PRRs	Interleukin-10	IL-10
Mitochondrial DNA	mtDNA	Mesenchymal stem cells	MSCs
Oxidative phosphorylation	OXPHOS	Extracellular matrix	ECM
Neutrophil extracellular traps	NETs	Matrix metalloproteinases	MMPs
Guideline-directed medical therapy	GDMT	Plant-derived exosome-like nanovesicles	PELNs
Cytotoxic T lymphocytes	CTLs	Reticuloendothelial system	RES
Regulated cell death	RCD	Nitric oxide	NO
Pan-apoptosis	PANoptosis	Extracellular vesicles	EVs
Gasdermin D	GSDMD	Astragaloside IV	AS-IV
Reactive oxygen species	ROS	Regulatory T cells	Tregs
Traditional Chinese Medicine	TCM	Induced pluripotent stem cell-derived cardiomyocytes	iPSC-CMs
Engineered Heart Tissues	EHTs	reticuloendothelial system	RES
Toll-like receptor 4	TLR4	Toll-like receptors	TLRs
T helper 17 cells	Th17	Interleukin-17	IL-17
Tumor necrosis factor alpha	TNF-α	Interleukin-18	IL-18

**Figure 1:**
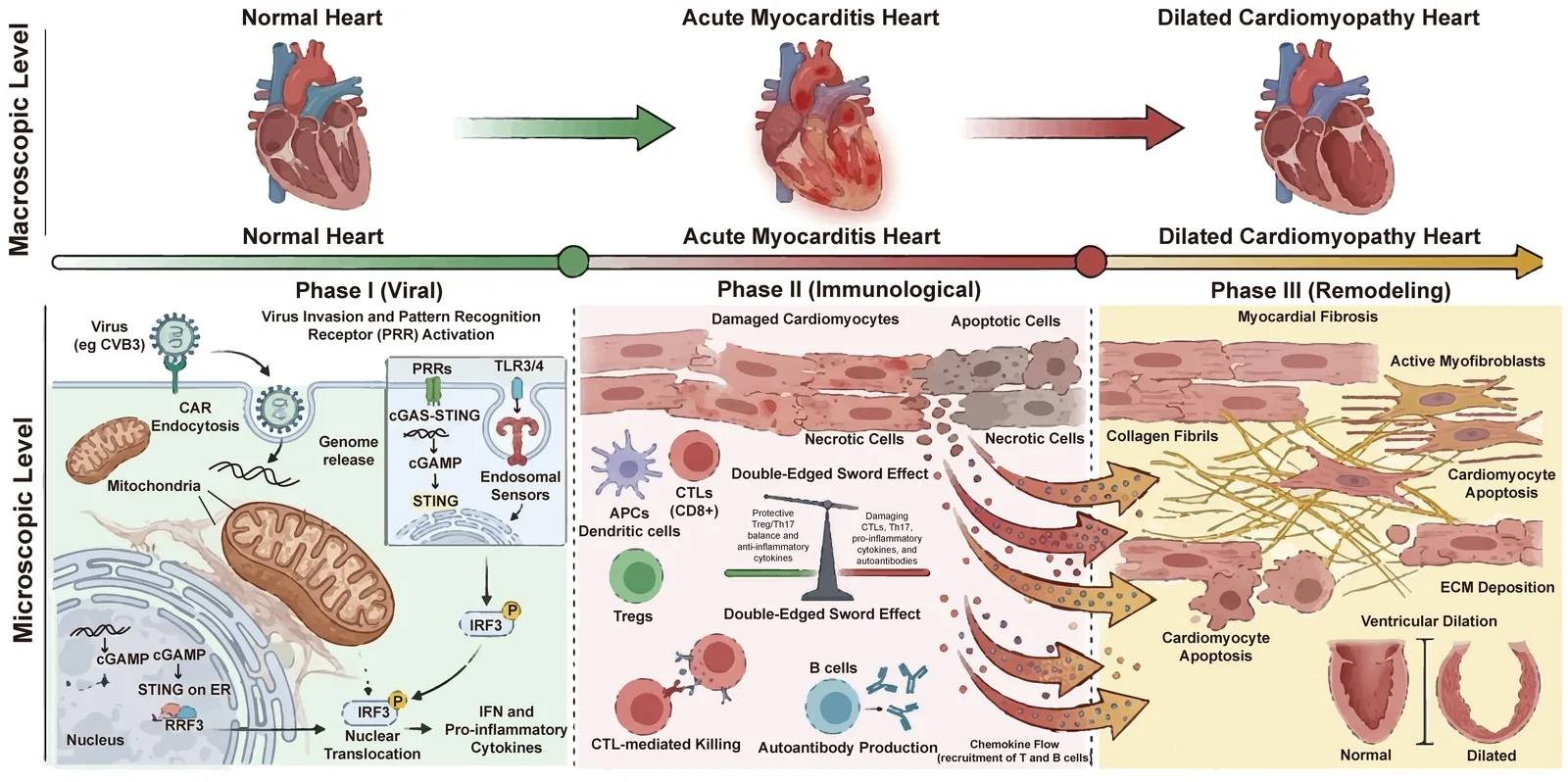
The triphasic pathophysiological cascade of viral myocarditis: from acute viral invasion to dilated cardiomyopathy. The schematic displays the tripartite progression of VMC: the initial viral phase involving direct viral lysis and PRR-mediated innate immune sensing; the subsequent immunological phase marked by adaptive immune cell infiltration and potential autoimmune cross-reactivity; and the final remodeling phase featuring progressive fibroblast activation, maladaptive fibrosis, and ventricular wall thinning. Created with Adobe Illustrator.

The transition from VMC to DCM is typically orchestrated by a well-defined, tripartite pathophysiological cascade of interlinked stages (**Figure 1**) (5). In the initial viral phase, cardiotropic viruses—most notably Coxsackievirus B3—infiltrate cardiac myocytes and undergo extensive replication, leading to direct cellular lysis. Concurrently, the host rapidly mobilizes an innate immune response mediated by pattern recognition receptors (PRRs), including TLRs, RIG-I, and inflammasomes, thereby establishing the primary line of defense (6). Subsequently, the immunological phase ensues, characterized by the massive infiltration of adaptive immune cells aimed at viral clearance. However, this response is frequently dysregulated; via mechanisms such as molecular mimicry and epitope spreading, the immune system may erroneously target endogenous cardiac proteins, thereby eliciting persistent autoimmune-mediated myocardial injury (5). Failure to resolve the inflammatory response in a timely manner drives the disease toward a chronic remodeling phase. This stage is marked by persistent low-grade inflammation, fibroblast activation, and maladaptive fibrosis; concurrently, the progressive attrition of cardiomyocytes compels structural ventricular remodeling, eventually culminating in the classic DCM phenotype (3, 7).

Within this highly dynamic microenvironment, the host immune response essentially functions as a double-edged sword: while acting as a vital defensive barrier to curtail viral dissemination, it simultaneously serves as a primary driver of detrimental myocardial structural remodeling (8). Traditional broad-spectrum immunosuppressive regimens, such as glucocorticoids, frequently fail to provide substantial clinical benefit due to their inherent lack of spatio-temporal specificity. While suppressing myocardial inflammation, these strategies concurrently compromise the host’s antiviral capacity, potentially exacerbating viral persistence (9, 10). Consequently, there is a compelling need for a paradigm shift in VMC treatment: transitioning from conventional systemic immunosuppression toward spatiotemporally precise immunomodulation. By leveraging smart biomaterials and nanomedicine, it is now feasible to selectively suppress autoimmune-mediated damage while maximizing the preservation of the host’s innate capacity for pathogen clearance.

## 2 Innate immunity: the first line of defence and collateral damage

Dysregulation of the innate immune response constitutes a pivotal pathological mechanism that drives the transition from early myocardial insult in VMC toward progressive heart failure.

### 2.1 Spatiotemporal regulation of pattern recognition receptors

PRR-mediated innate immune responses in VMC exhibit distinct spatiotemporal characteristics. During the acute phase of infection, Toll-like receptor 3 (TLR3) functions as a critical sensor for double-stranded RNA (dsRNA), exerting a protective role by inducing the expression of Type I interferons (IFNs) to establish a robust antiviral state (11, 12). However, as the disease progresses and myocardial injury ensues, the Toll-like receptor 4 (TLR4) signaling pathway is extensively triggered by damage-associated molecular patterns (DAMPs), such as HMGB1, released from the necrotic myocardium. This shift causes the initial defense mechanism to maladaptively transform into a primary driver of uncontrolled sterile inflammation and secondary myocardial damage (13, 14).

**Figure 2:**
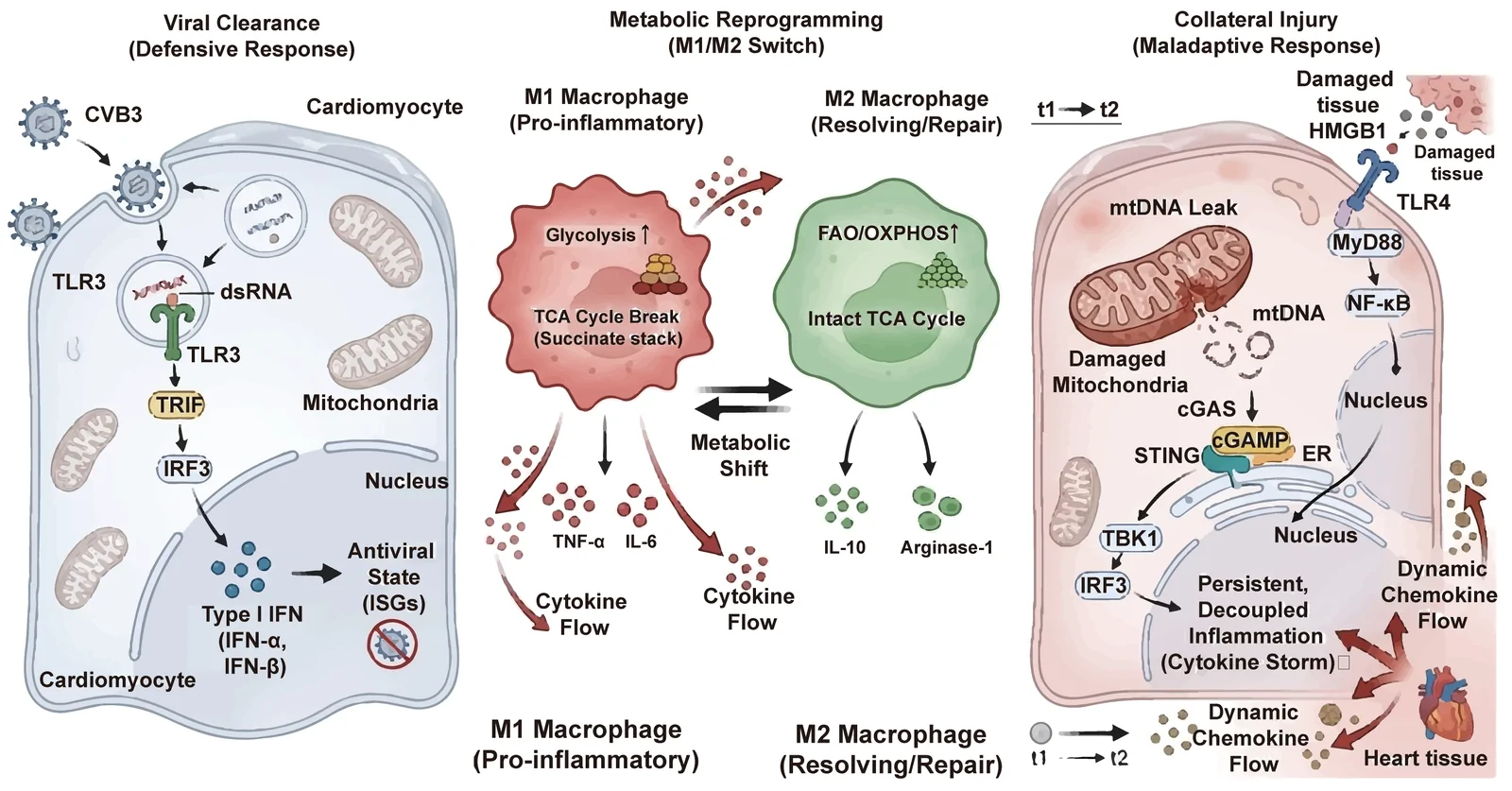
Innate immune sentinels and metabolic reprogramming: balancing viral clearance and collateral myocardial injury. The diagram displays: the defensive viral clearance phase, involving dsRNA recognition through the TLR3–TRIF–IRF3 cascade to drive Type I interferon and ISG production; the metabolic reprogramming phase, marked by the M1-to-M2 macrophage switch, where a transition from a glycolytic TCA cycle break (succinate stack) to intact FAO/OXPHOS shifts cytokine flow toward repair; and the maladaptive collateral injury phase, featuring persistent inflammation and cytokine storms driven simultaneously by HMGB1/TLR4 signaling and mtDNA leak-mediated cGAS–STING activation. Created with Adobe Illustrator.

Furthermore, the cGAS-STING axis serves as a pivotal molecular bridge that couples cellular stress with persistent autoimmunity. Mitochondrial impairment secondary to RNA viral infection triggers the translocation of mitochondrial DNA (mtDNA) into the cytosol, where it is misidentified by cGAS as a pathogenic threat. This aberrant sensing initiates a robust pro-inflammatory cascade that fosters a ‘decoupled’ state of immune activation—one that endures beyond viral eradication and inexorably drives the progression toward chronic inflammatory cardiomyopathy (**Figure 2**) (15, 16).

### 2.2 Metabolic reprogramming and phenotypic rigidity in macrophages

The functional polarization of macrophages in the VMC microenvironment is intricately linked to their underlying metabolic reprogramming. During the acute inflammatory phase, macrophages undergo a metabolic shift toward aerobic glycolysis, fueling the pro-inflammatory M1 phenotype required for initial viral control. Conversely, the resolution phase necessitates a transition to mitochondrial oxidative phosphorylation (OXPHOS) to drive the switch toward the pro-repair M2 phenotype. Disruption of this metabolic ‘toggle’ traps macrophages in a persistent M1 state, thereby precipitating chronic, unresolved inflammation (17–19).

At the heart of this sustained injury lies the recruitment of infiltrating CCR2^+^ monocyte-derived macrophages, a population that proves particularly deleterious to myocardial stability. While resident cardiac macrophages typically preserve local immune tolerance under homeostatic conditions, the stress of viral infection triggers a massive influx of peripheral CCR2^+^ counterparts. These cells exhibit profound ‘phenotypic rigidity’—a resistant state that defies repolarization toward an anti-inflammatory M2 phenotype. By functioning as a pathogenic hub, their persistent secretion of cytotoxic mediators directly executes cardiomyocyte apoptosis, ultimately shattering local homeostasis and precipitating irreversible ventricular remodeling (20, 21).

### 2.3 The double-edged sword effect of neutrophil extracellular traps

Although NETs released during the initial stages of infection serve as a physical barrier to sequester viral dissemination, their aberrant formation can precipitate profound pathological collateral damage. These unregulated extracellular DNA scaffolds facilitate platelet aggregation, thereby eliciting immunothrombosis within the myocardial microvasculature and further aggravating local ischaemia and hypoxia (22). Concurrently, their abundant histone components can directly compromise the integrity of myocardial cell membranes, thereby precipitating a deleterious positive feedback loop of “inflammation–tissue damage (23).

## 3 Adaptive immunity: the boundary between specificity and autoimmunity

**Figure 3:**
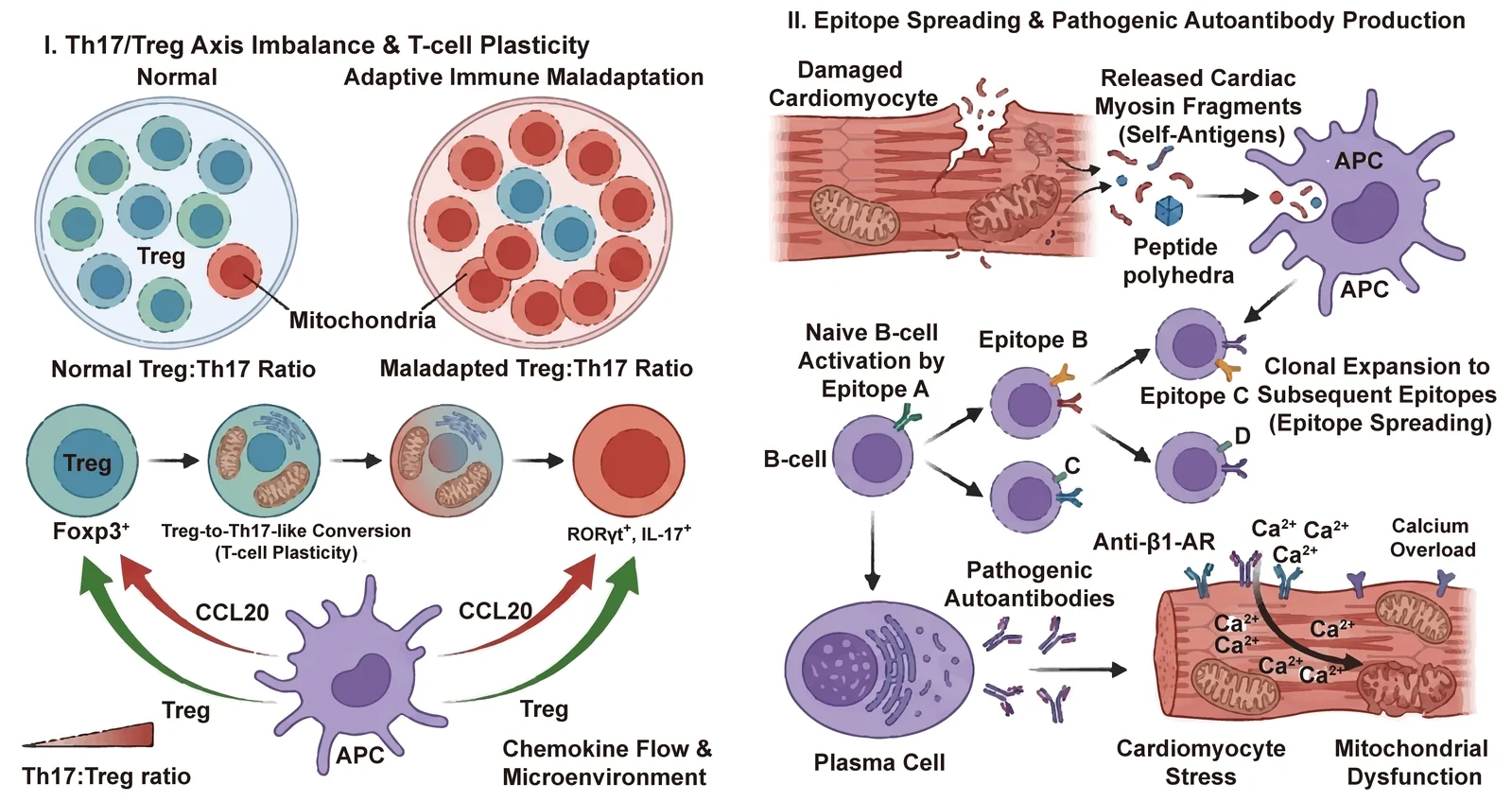
Adaptive immune maladaptation: T-cell plasticity, epitope spreading, and pathogenic autoantibody production. This schematic highlights the adaptive immune dysregulation, focusing on the loss of balance between Th17 and Treg populations and the consequent T-cell plasticity. It also demonstrates the B-cell-mediated humoral response, where the release of self-antigens from damaged cardiomyocytes triggers epitope spreading and the production of functional, pathogenic autoantibodies that contribute to sustained myocardial injury. Created with Adobe Illustrator.

Subsequent to innate immune activation, the recruitment of adaptive immune cells marks a critical inflection point in VMC pathogenesis. At this stage, the interplay between protective antiviral immunity and detrimental autoimmune responses emerges as the principal driver of chronic myocarditis and its progression to DCM (**Figure 3**).

### 3.1 Plasticity and pathogenicity of T cells

Amidst the sustained inflammatory milieu of VMC, the immunological equilibrium between pro-inflammatory T helper 17 cells (Th17) and immunosuppressive Treg populations is perturbed. This consequent dysregulation of the Th17/Treg ratio serves as a pivotal factor dictating the trajectory of myocardial inflammation toward either resolution or chronicity (24). Specifically, Interleukin-17 (IL-17) secreted by Th17 cells not only orchestrates the recruitment of inflammatory cells to sustain a milieu of local hyperinflammation but also directly promotes cardiac fibroblast activation via the induction of matrix metalloproteinases (MMPs). Consequently, this mechanism shifts the role of the Th17 response from a proinflammatory initiator to a pivotal driver of myocardial fibrosis and structural remodeling (25). Concurrently, the stability of the Treg population is compromised. While Tregs are pivotal for immune regulation during the acute phase of VMC, the persistent high-level cytokine milieu—particularly interleukin-6(IL-6)—induces significant phenotypic plasticity, driving the transdifferentiation of Tregs into Th17-like cells. This collapse of immune tolerance subsequently exacerbates immune-mediated myocardial injury (26, 27).

Furthermore, bystander tissue injury mediated by CD8+ cytotoxic T lymphocytes (CTLs) represents a critical driver of disease progression. While CTLs primarily facilitate viral clearance through the precise execution of the perforin-granzyme B pathway, their excessive or indiscriminate activation can inadvertently precipitate significant collateral damage to neighboring healthy cardiomyocytes (28, 29). However, in the setting of fulminant myocarditis, hyperactivated CTLs undergo an unchecked cytotoxic transition, inflicting profound bystander injury upon uninfected cardiomyocytes prior to viral clearance. This aberrant immune-mediated destruction serves as a primary precipitant of acute heart failure and lethal arrhythmias (30).

### 3.2 B cells and humoral immunity: from epitopes to functional antibodies

Beyond their canonical function as antigen-presenting cells, B cells actively contribute to myocardial injury through a distinct pathogenic process: the production of autoreactive antibodies. This humoral response serves as a critical, non-canonical driver of disease progression in myocarditis.

Within the framework of adaptive humoral immunity, B-cell-driven autoimmune feedback loops represent a pivotal mechanism underpinning the transition to chronic disease. Subsequent to the acute phase of viral infection, autoantigens (e.g., cardiac myosin) released upon cardiomyocyte lysis are sequestered within the inflamed myocardial milieu, facilitating epitope spreading among B-cell subsets. This process culminates in a robust, secondary autoimmune attack directed against host cardiac tissue, thereby sustaining chronic myocardial damage (31). This process causes the inflammatory response to gradually dissociate from the initial viral trigger, becoming autonomously driven by the autoimmune circuit, thereby providing an explanation for the pathogenic mechanism underlying the clinical phenomenon of ‘persistent inflammation despite viral negativity’ (32).

Furthermore, the receptor-modulating effects of functional autoantibodies further exacerbate irreversible myocardial damage. Functional autoantibodies with agonist-like activity, such as anti-β1-AR antibodies, can lead to intracellular calcium overload and mitochondrial dysfunction in cardiomyocytes through sustained activation of the cAMP-PKA pathway (33). This immune-mediated receptor overstimulation can induce sustained cardiomyocyte loss and interstitial fibrosis even in the absence of significant inflammatory cell infiltration, thereby significantly accelerating the progression of decompensated heart failure.

## 4 Cross-talk and the mechanisms of cell death

**Figure 4:**
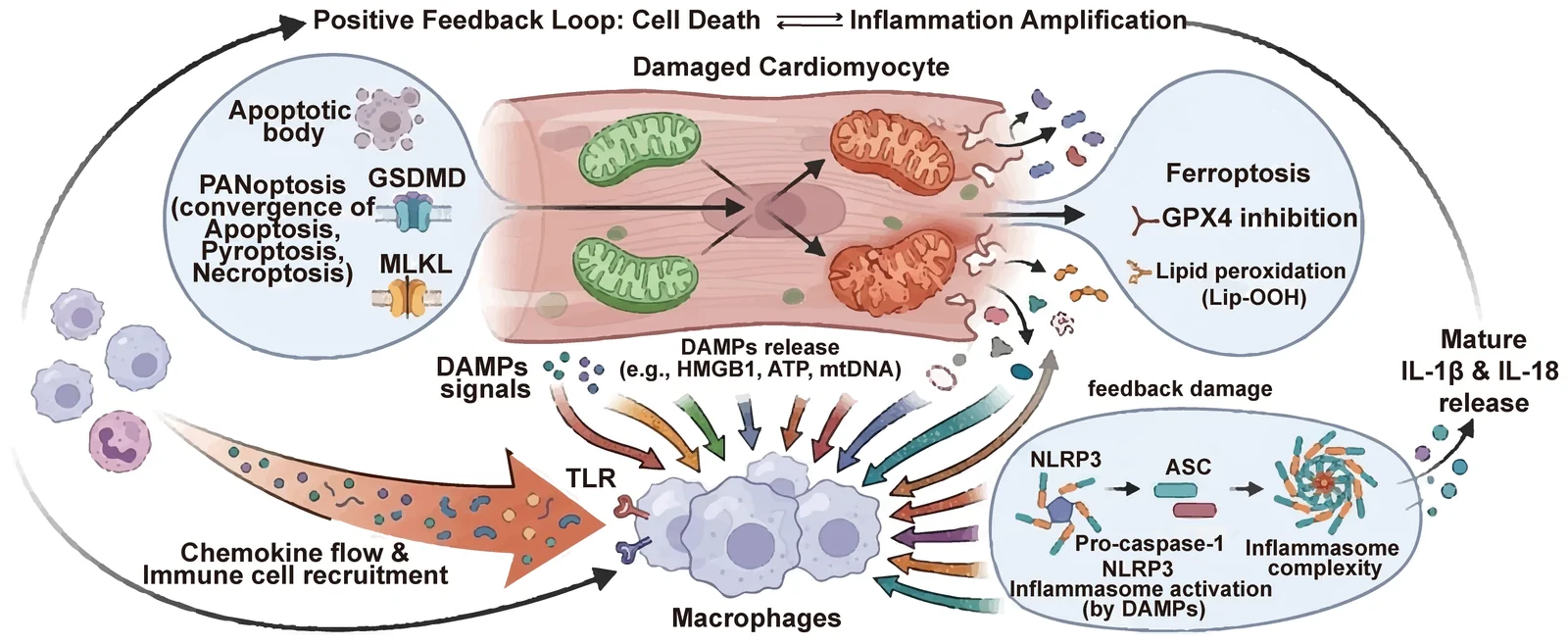
Cross-talk between cardiomyocyte programmed cell death and immune amplification: the role of PANoptosi:and ferroptosis. This figure illustrates the complex interplay between regulated cell death pathways and innate immune activation. It highlights how the convergence of apoptosis, pyroptosis, and necroptosis (PANoptosis) alongside ferroptosis leads to the release of DAMPs, which in turn drive the assembly of the NLRP3 inflammasome, creating a positive feedback loop of sustained inflammation and myocardial damage. Created with Adobe Illustrator.

The progression of VMC is not merely a consequence of viral replication; its fundamental driving force lies in the intricate crosstalk between damaged cardiac muscle cells and the immune system. RCD does not occur in isolation, but acts as a potent immunogenic signal that determines the intensity and duration of the inflammatory response (**Figure 4**).

### 4.1 Pan-apoptosis and the NLRP3 inflammasome

Although it is traditionally believed that classical apoptosis does not trigger inflammation, in the context of VMC, myocardial cell death manifests as a ‘pan-apoptosis’ pattern that integrates pyroptosis, apoptosis and necrotic apoptosis.

Within the highly cross-regulated programme of PANoptosis, the NLRP3 inflammasome plays a pivotal role as a central hub. Specifically, viral infection and the microenvironmental stress it induces can directly trigger the assembly of the NLRP3 inflammasome and activate Caspase-1, thereby leading to the maturation and release of the pro-inflammatory cytokines Interleukin-1β (IL-1β) and Interleukin-18 (IL-18) (34). Subsequently, activated Caspase-1 further cleaves Gasdermin D (GSDMD) to induce pyroptosis, thereby driving the formation of a positive feedback loop of inflammation. This mode of cell death, characterised by the disruption of membrane integrity, releases large quantities of DAMPs into the extracellular space; these act as potent immunogenic signals capable of rapidly recruiting and activating neutrophils and macrophages (35, 36). It is thus evident that pyroptosis in cardiomyocytes is not merely the result of local inflammatory attacks, but also a positive feedback trigger that leads to the sustained propagation of the inflammatory cascade.

### 4.2 Immunogenic ferroptosis

As cardiomyocytes are highly dependent on mitochondrial metabolism and rich in polyunsaturated fatty acids, they are naturally susceptible to ferroptosis. During the metabolic collapse and inflammatory amplification in the acute phase of VMC, intense oxidative stress significantly inhibits GPX4 activity, leading to the massive accumulation of toxic lipid reactive oxygen species (ROS), which in turn triggers cellular ferroptosis (37). As ferroptosis occurs, the autoimmune response is further exacerbated. DAMPs and oxidised lipid signals released upon the lysis of damaged cardiomyocytes not only directly drive macrophage polarisation towards the pro-inflammatory M1 phenotype, but also further promote the activation of CTLs (38, 39). This highly immunogenic microenvironment shifts the body’s immune surveillance focus from the initial clearance of the virus to a sustained immune attack on the damaged myocardium, ultimately establishing a local pathological axis of ‘metabolic collapse—inflammation amplification—autoimmunity exacerbation’ that drives disease progression.

### 4.3 Mitochondrial dysfunction: a molecular hub for immune amplification

Mitochondria are not only the metabolic centres of the cell, but also a key hub for integrating metabolic status with innate immune signalling. Under the stress of viral invasion, mitochondrial membrane permeability in cardiomyocytes increases significantly, leading to the leakage of mtDNA into the cytoplasm. This ectopic mtDNA is recognised by cGAS, which subsequently activates the cGAS-STING pathway and drives the robust expression of type I IFNs and pro-inflammatory factors, thereby rapidly transforming local metabolic stress into a systemic inflammatory amplification response (40, 41). Concurrently, excessive mitochondrial ROS generated by damaged mitochondria play a key pathogenic role; they can directly trigger the assembly of the NLRP3 inflammasome and significantly enhance the maturation of IL-1β (42). Taken together, this intricate ‘mitochondrial-immune axis’ ensures that myocardial tissue continues to provide endogenous danger signals even after viral load has declined, ultimately driving the irreversible malignant transformation of VMC into dilated cardiomyopathy (DCM).

## 5 Therapeutic strategies: from systemic suppression to precision bioengineering

### 5.1 Limitations and optimisation of current clinical immunological interventions

Before exploring emerging bioengineering paradigms, it is necessary to first assess the current state of pharmacological interventions. Whilst guideline-directed medical therapy (GDMT) addresses haemodynamic instability, specific immunomodulatory therapies are designed to tackle the underlying causes—namely, persistent viral infection and immune hyperreactivity. However, these strategies are often limited by a narrow therapeutic window and systemic side effects.

#### 5.1.1 Immunomodulatory therapy

In the clinical management of VMC, immunomodulatory therapies aim to suppress harmful inflammatory responses whilst preserving the host’s ability to defend against the virus as much as possible. Among these, intravenous immunoglobulin (IVIG) remains one of the most commonly used—and most controversial—interventions, and is widely employed particularly in paediatric patients and cases of fulminant myocarditis (43, 44).

The potential therapeutic effects of IVIG are thought to stem from its multifaceted immunomodulatory mechanisms. Firstly, IVIG can directly bind to viral particles or toxins via the neutralising antibodies it contains, thereby reducing viral load and limiting its dissemination to tissues (9). Secondly, IVIG can saturate Fcγ receptors on the surface of monocytes, macrophages and natural killer cells, thereby inhibiting antibody-dependent cellular cytotoxicity (ADCC) and alleviating immune-mediated myocardial damage (45). Furthermore, IVIG can limit the inflammatory amplification and tissue damage mediated by the complement cascade by blocking complement activation and the generation of C3/C5 cleavage products (46). Although IVIG offers extensive immunomodulatory benefits in theory and in experimental settings, its clinical efficacy in VMC exhibits marked heterogeneity. Randomised controlled trials and systematic reviews indicate that IVIG does not consistently improve survival or left ventricular function in all adult patients with VMC (47). This variation suggests that the benefits of IVIG may be highly dependent on the disease stage and aetiological background: in patients during the acute viremia phase or whilst viral replication remains active, its antiviral and immunomodulatory effects may be more pronounced, whereas its efficacy is markedly limited in later stages dominated by immune-mediated damage (44). Furthermore, some studies suggest that IVIG may yield higher response rates in infections with specific viral subtypes (such as parvovirus B19), indicating that IVIG may be more effectively targeted at specific patient groups rather than administered to all patients (48).

Unlike IVIG, interferon therapy directly targets the initial viral triggers in the pathogenesis of VMC, representing an immunomodulatory strategy centred on enhancing the host’s antiviral capacity (49). Exogenous IFNs, particularly interferon-β (IFN-β), can enhance the degradation of viral RNA/DNA and inhibit viral replication by upregulating the expression of interferon-stimulated genes (ISGs), thereby promoting the clearance of the viral genome (50). In patients with VMC and persistent viral infection, IFN-β treatment has been shown to reduce myocardial viral load and improve left ventricular function (51).

However, a key limitation of interferon therapy lies in its marked time-dependence. Whilst interferon signalling exerts a clear protective effect in the early stages of infection, as the disease progresses to the immune-mediated phase, a sustained or exogenously enhanced interferon response may amplify the inflammatory cascade and even exacerbate myocardial damage (52, 53). Consequently, interferon therapy is more suitable for patients in whom viral replication remains detectable, whereas its use must be approached with great caution during the virus-negative phase, which is primarily driven by autoimmunity or inflammation (54).

In summary, both IVIG and interferon therapy embody the core principles of immunomodulation in VMC treatment; however, their efficacy is highly dependent on the precise timing of administration and pathogen stratification. This fact further underscores the importance of pathogen detection and immunophenotyping in VMC, and lays the foundation for future mechanism-based, personalised immunomodulatory strategies (55).

#### 5.1.2 Biologic agents targeting inflammatory pathways

As our understanding of the immunopathological mechanisms underlying VMC continues to deepen, biologics targeting key inflammatory factors are increasingly being regarded as an attractive therapeutic strategy; the core concept lies in alleviating immune-mediated myocardial damage by blocking specific inflammatory cascades (56). Against this backdrop, pro-inflammatory cytokines such as IL-1β and IL-6 have emerged as key targets for intervention due to their central role in amplifying inflammation and exacerbating myocardial dysfunction (57).

Canakinumab is a fully humanised monoclonal antibody that targets IL-1β; its cardiovascular protective effects were first demonstrated by the groundbreaking findings of the Canakinumab Anti-inflammatory Thrombosis Outcomes Study (CANTOS) trial. This study showed that inhibiting IL-1β-mediated inflammatory signalling significantly reduced the incidence of major adverse cardiovascular events without affecting lipid levels (58, 59). Given the role of IL-1β in mediating myocardial dysfunction and amplifying inflammatory signals during the acute phase of VMC, canakinumab is considered to have a plausible mechanism of action for inhibiting immune-mediated myocardial injury during the inflammatory amplification phase (60).

Another class of biologics attracting widespread attention are IL-6 pathway inhibitors; among these, tocilizumab—a monoclonal antibody that blocks the IL-6 receptor—was widely used during the COVID-19 pandemic to treat cytokine release syndrome, demonstrating its value in controlling severe inflammatory states and improving clinical outcomes (61). Given that fulminant myocarditis is often accompanied by markedly elevated IL-6 levels and a systemic inflammatory response, tocilizumab is currently being used on an exploratory basis in this patient population, with the aim of alleviating the inflammatory response, stabilising haemodynamics and limiting further myocardial damage by blocking IL-6 signalling (62).

However, even these highly targeted biologics face significant challenges in clinical application. Firstly, systemic administration lacks tissue specificity, making it difficult to distinguish between harmful inflammation and necessary antiviral immune responses, thereby exposing patients to the risk of widespread immunosuppression (63). Secondly, IL-1β and IL-6 signalling also play physiological roles in host defence and tissue repair; their long-term or excessive suppression may increase the risk of opportunistic infections and reactivation of latent viruses (64).

Therefore, current evidence suggests that the use of biologics in VMC is more likely to be appropriate for a specific subgroup of patients with highly active inflammation and controlled viral replication, rather than as a universal treatment (51). This reality also highlights the urgent need for a new generation of therapeutic strategies—namely, the development of precision delivery systems capable of specifically concentrating anti-inflammatory drugs within inflamed myocardial tissue, thereby maximising the suppression of pathological inflammation whilst minimising the adverse consequences of systemic immunosuppression (65).

### 5.2 Emerging biomaterials and nanomedicine applications (emerging bioengineering and nanomedicine strategies)

Although immunomodulatory drugs and biologics have shown some promise in the treatment of VMC, their clinical application is often limited by factors such as limited bioavailability, short half-lives *in vivo*, and significant off-target effects (66). Particularly in VMC—a highly dynamic and stage-dependent disease—systemic administration struggles to suppress pathological inflammation whilst preserving the necessary antiviral immune response, thereby limiting the efficacy and safety of conventional treatment strategies (67).

**Figure 5:**
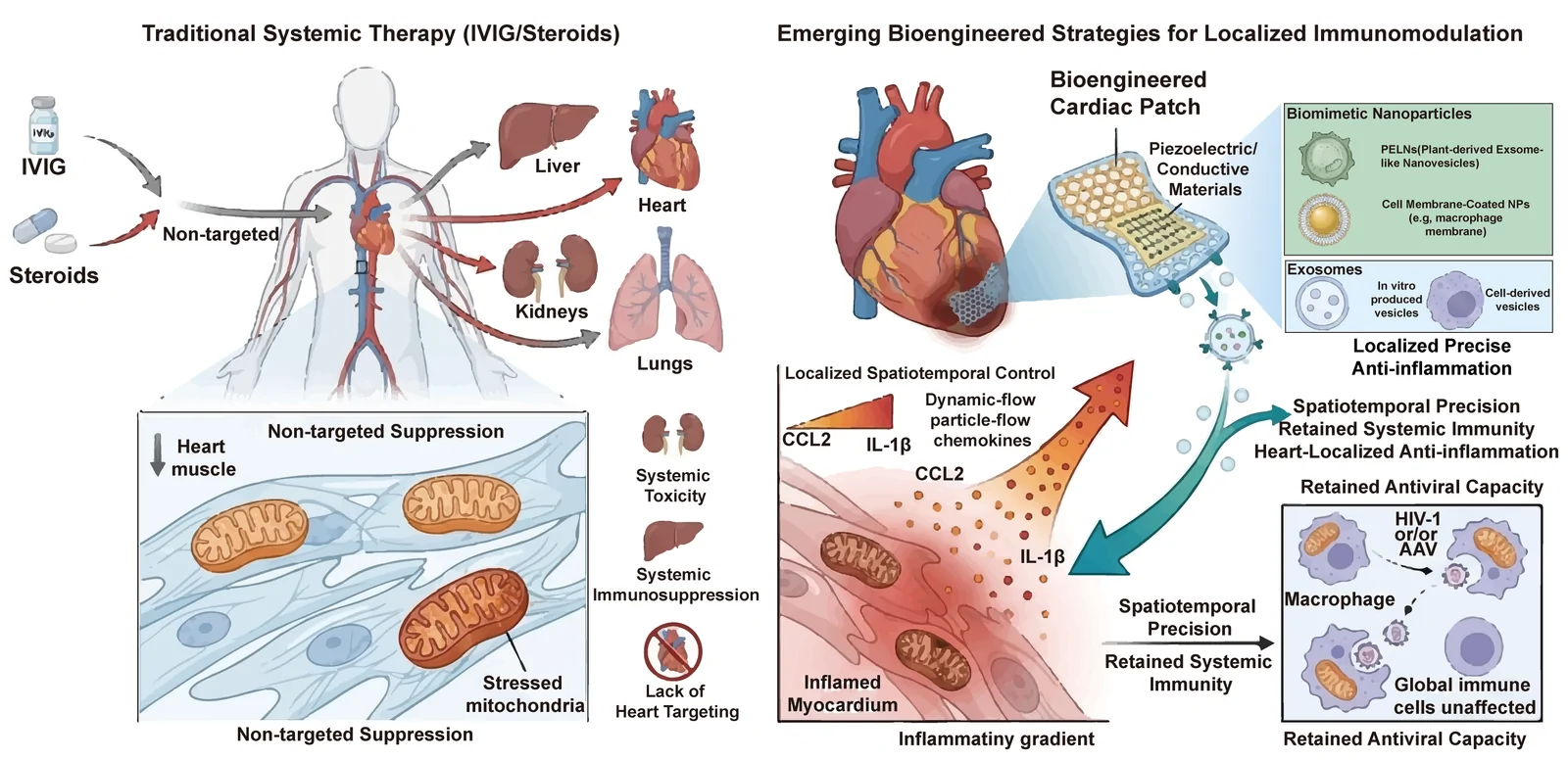
Paradigmatic shift in therapeutic strategies: transitioning from systemic immunosuppression to bioengineered precision immunomodulation this schematic contrasts the limitations of traditional non-targeted systemic immunosuppressive therapies with emerging bioengineering strategies. It demonstrates how localized delivery platforms, such as cardiac patches, exosomes, plant-derived exosome-like nanovesicles (PELNs), biomimetic nanoparticles, and piezoelectric devices, can provide spatiotemporal regulation of the immune microenvironment, effectively suppressing pathological inflammation while preserving host antiviral capacity. Created with Adobe Illustrator.

With the rapid advancement of bioengineering and materials science, novel biomaterials that integrate precise delivery, controlled release and responsiveness to the immune microenvironment are opening up entirely new therapeutic avenues for VMC. Compared with conventional drugs, these engineered materials enable fine-tuned regulation of drug action in both time and space, allowing therapeutic molecules to be preferentially concentrated in damaged myocardial regions, thereby significantly enhancing local efficacy and reducing systemic toxicity (68). This concept of ‘localised, precise immune modulation’ aligns perfectly with the urgent need for stage-specific intervention during the progression of VMC from acute inflammation to chronic inflammatory cardiomyopathy and ultimately to DCM (**Figure 5**) (69).

Against this backdrop, a range of emerging bioengineering strategies have begun to attract attention, including cardiac patches for mechanical support and immune regulation, smart biomaterials with anti-inflammatory or immunomodulatory properties, and biomimetic nanomedicine systems that mimic the characteristics of natural cells or exosomes (70, 71). These strategies not only serve as drug delivery vehicles but also reshape the local inflammatory microenvironment through direct interaction with immune cells, thereby intervening in immune-mediated myocardial injury at the mechanistic level (72).

The introduction of novel biomaterials and nanomedicine marks a shift in VMC treatment from ‘systemic immunosuppression’ to ‘tissue-specific immune reprogramming’ (73). To provide specific examples of these emerging bioengineering strategies, several advanced biomaterial platforms have demonstrated profound efficacy in targeted cardiovascular repair and immunomodulation. Beyond patches and hydrogels, the surface biofunctionalization of cardiovascular scaffolds (such as polycaprolactone and other elastic polymers) with polyzwitterions or extracellular matrix components actively prevents thrombosis and promotes rapid endothelialization (74). These advanced vascular scaffolds can also be integrated with flexible bioelectronics for the intelligent, real-time monitoring of hemodynamics and restenosis (74). By combining engineering innovations with an understanding of immune mechanisms, this field holds promise for providing more precise and sustainable interventions to prevent the progression of VMC to DCM, whilst also laying a vital foundation for the realisation of future personalised treatment strategies (73). Collectively, these specific biomaterial applications exemplify the transition towards tissue-specific immune reprogramming and precision intervention in inflammatory cardiovascular diseases.

#### 5.2.1 Functional cardiac patches and hydrogels: *in situ* microenvironment remodelling

Cardiac patches and hydrogels are fundamental biomaterial platforms for localized cardiovascular intervention (75, 76). Cardiac patches are engineered planar constructs applied to the epicardial surface to provide mechanical reinforcement to the injured ventricular wall and serve as reservoirs for targeted drug or cell delivery (77, 78). Hydrogels are highly hydrated, three-dimensional polymeric networks that biomimetically resemble the natural extracellular matrix (ECM) (79).With tunable rheological properties such as shear-thinning and thermosensitivity, hydrogels can either be administered via minimally invasive intramyocardial injection or utilized as the structural scaffold for cardiac patches (80, 81).As the regenerative capacity of adult cardiomyocytes is extremely limited, the structural damage and immune dysregulation resulting from VMC are often difficult to fully reverse through endogenous repair mechanisms (82). Against this backdrop, functionalised hydrogels and cardiac patches have gradually evolved from traditional ‘mechanical scaffolds’ into active therapeutic platforms that integrate structural support, immune modulation and biosignal delivery (83, 84). Through *in situ* implantation or local injection, these engineered materials can act directly on damaged myocardial regions, thereby reshaping the local microenvironment—where inflammation and repair coexist—in both spatial and temporal dimensions (68). For instance, Engineered Heart Tissues (EHTs) constructed from collagen or fibrin hydrogels encapsulating induced pluripotent stem cell-derived cardiomyocytes (iPSC-CMs) have been successfully deployed as specific cardiac patches to remuscularize infarcted tissues and restore cardiac function (85).

A key advantage is that hydrogels and cardiac patches can store immunomodulators, enabling the sustained, controlled release of therapeutic molecules (86). By encapsulating anti-inflammatory cytokines (such as interleukin-10, IL-10), mesenchymal stem cells (MSCs) or their derived exosomes within thermosensitive or photo-crosslinked hydrogels, a stable paracrine signalling gradient can be established at the site of the lesion (87, 88). This local delivery strategy not only significantly increases the effective biological concentration but also avoids the widespread immunosuppression and risk of infection associated with systemic administration (89). In the realm of injectable systems, shear-thinning and self-healing hydrogels engineered with specific tethered bioactive cues (e.g., SDF-1α, substance P, or IGF-1C) significantly enhance the localized retention of therapeutic stem cells and extracellular vesicles, effectively modulating the hostile inflammatory microenvironment (90). Moreover, nitric oxide (NO)-eluting hydrogels and metal-catechol-amine network coatings provide spatiotemporally precise immunomodulation by competitively promoting endothelial cell adhesion while suppressing pathological smooth muscle cell proliferation and local inflammation (91). In the VMC model, such paracrine signals have been shown to induce a shift in the phenotype of infiltrating macrophages and T cells from a pro-inflammatory to a pro-repair and immunomodulatory phenotype, thereby accelerating the resolution of inflammation and promoting tissue remodelling (92, 93).

In addition to biochemical regulation, the role of smart biomaterials at the mechanical level should not be overlooked. Various hydrogels and cardiac patches have been designed to possess viscoelastic properties similar to those of the natural extracellular matrix (ECM), enabling them to match the dynamic contraction-relaxation behaviour of cardiac muscle (94). Mechanistically, these biomaterials reduce myocardial wall stress through dual biomechanical pathways. Injectable hydrogels undergo *in situ* gelation to increase ventricular wall thickness and decrease its local radius of curvature, thereby directly reducing diastolic wall stress in accordance with Laplace’s law (95). Meanwhile, epicardial patches with anisotropic tensile properties act as external elastic constraints (96).They physically resist diastolic overstretching and redistribute pathological mechanical loads. This mechanical offloading directly blunts stretch-activated mechanotransduction pathways in cardiac fibroblasts, suppressing maladaptive pro-fibrotic signaling at the source (97, 98).In the late stages of VMC or the early stages of transition to DCM, implanted cardiac patches can act as ‘elastic constraints’, providing the necessary mechanical tension support for thinned and dilated ventricular walls (99). By physically restricting pathological ventricular dilation, such materials can reduce ventricular wall stress, improve the distribution of mechanical load on the myocardium, and indirectly inhibit excessive fibroblast activation and the fibrotic process (100, 101). In experimental models of viral myocarditis, localized application of these biomaterials yields distinct therapeutic benefits (102). Functionally, they preserve myocardial contractility, maintaining left ventricular ejection fraction (LVEF) and fractional shortening (FS). Histologically, they attenuate collagen deposition and prevent adverse ventricular remodeling (103). The synergistic strategy of mechanical offloading and immunomodulation has proven highly effective in clinical settings (e.g., using mechanical circulatory support and systemic immunosuppressants) (104). By recapitulating this synergy locally, smart biomaterials hold significant promise in mitigating the progression from acute VMC to DCM and heart failure. Furthermore, gelatin-based microneedle patches enable the deep-tissue delivery of exosome-loaded hydrogels to attenuate fibrosis (85).

Smart cardiac patches and functionalised hydrogels offer an intervention strategy distinct from conventional pharmacological treatments by simultaneously targeting the immune microenvironment and the cardiac mechanical structure. This concept of *in situ* microenvironmental remodelling transforms biomaterials from passive carriers of tissue repair into therapeutic tools that actively regulate the inflammation–repair balance, thereby providing a highly promising engineering solution for preventing the progression of VMC to DCM (**Table 2**) (105).

**Table 2:** Summary of functional cardiac patches and hydrogels for *in situ* microenvironment remodelling.

Biomaterial system	Bioactive cue	Significant findings	References
Engineered Heart Tissues (EHTs)/Cardiac patches	iPSC-CMs	Remuscularize infarcted tissues Restore cardiac function	(85)
Thermosensitive or photo-crosslinked hydrogels	Anti-inflammatory agents	Establish a stable paracrine signalling gradient Induce a shift of macrophages and T cells to a pro-repair/immunomodulatory phenotype Accelerate inflammation resolution	(87, 88, 92, 93)
Self-healing hydrogels	Tethered bioactive cues	Significantly enhance the localized retention of therapeutic stem cells and extracellular vesicles Effectively modulate the hostile inflammatory microenvironment	(90)
Hydrogels and network coatings	Nitric oxide (NO)	Provide spatiotemporally precise immunomodulation Promote endothelial cell adhesion Suppress pathological smooth muscle cell proliferation and inflammation	(91)
Biomimetic viscoelastic hydrogels and cardiac patches	Intrinsic mechanical properties	Act as ‘elastic constraints’ providing mechanical support Reduce ventricular wall stress and improve mechanical load distribution Indirectly inhibit excessive fibroblast activation and fibrosis	(94, 99–101)
Gelatin-based microneedle patches	Exosome-loaded hydrogels	Enable the deep-tissue delivery of therapeutic exosomes Attenuate myocardial fibrosis	(85)

EHTs, engineered heart tissues; iPSC-CMs, induced pluripotent stem cell-derived cardiomyocytes; NO, nitric oxide.

#### 5.2.2 Piezoelectric and conductive materials: mechano-electrical coupling for immunomodulation

The use of the mechanical energy generated by the heart’s own beating for therapeutic purposes has been a major innovation in recent years. Piezoelectric materials (such as PVDF nanofibres) possess the ability to convert mechanical stress into electrical signals. Specifically, during the rhythmic systole and diastole of the cardiac cycle, the implanted piezoelectric scaffold undergoes cyclic mechanical deformation. This continuous macroscopic strain induces an asymmetric shift in the internal dipole moments of the piezoelectric matrix, driving the accumulation of surface charges (106, 107). Consequently, a localized, strain-responsive microelectric field is established, delivering physiological-level microcurrents synchronously with the intrinsic heartbeat without requiring external power sources. Firstly, moderate electrical stimulation has been shown to promote the polarisation of M1 macrophages into M2 (anti-inflammatory/repair) macrophages by modulating intracellular calcium ion signalling pathways, thereby alleviating the inflammatory response (108). Secondly, conductive materials (such as carbon nanotubes and matrices doped with gold nanoparticles) can re-establish electrical connections between damaged myocardial tissues, improve the speed of electrical conduction in the affected areas, and significantly reduce the incidence of VMC-related malignant arrhythmias (109). Building on this, intrinsically functional biomaterials, such as polypyrrole-based conductive fibers or magnetic cardiac patches, actively restore electrical conduction and recruit endothelial cells (85). Furthermore, recent innovations have introduced optoelectronically active scaffolds, such as 3D-printed methacrylated gelatin blended with micro-solar cells, which can synchronize with the host myocardium and modulate the beating of the heart via pulsed light, thereby offering a novel strategy to prevent pro-arrhythmogenic risks (85). Collectively, the integration of piezoelectric and conductive properties establishes a synergistic mechano-electrical feedback loop within the inflamed myocardium. Piezoelectric elements harness intrinsic cardiac mechanics to generate localized electrical cues, directly driving anti-inflammatory macrophage repolarization to intervene in the innate immune response (110, 111). Simultaneously, conductive networks facilitate the uniform propagation of these immunomodulatory signals across the damaged tissue while stabilizing global cardiac electrophysiology (112, 113). This seamless coupling of electroactive structural restoration and localized immune reprogramming effectively addresses both the arrhythmogenic and inflammatory substrates of viral myocarditis (**Table 3**).

**Table 3:** Overview of electroactive and optoelectronic biomaterials for cardiac immunomodulation and electrical restoration.

Biomaterial system	Bioactive cue	Significant findings	References
Piezoelectric materials	PVDF nanofibres	Convert mechanical stress into electrical signals to mimic physiological microcurrents Promote M1 to M2 macrophage polarization via intracellular calcium signalling Alleviate the inflammatory response	(108, 198)
Conductive materials	Carbon nanotubes and gold nanoparticles	Re-establish electrical connections between damaged myocardial tissues Improve conduction speed Significantly reduce the incidence of VMC-related malignant arrhythmias	(109)
Intrinsically functional biomaterials	Polypyrrole-based conductive fibers or magnetic cardiac patches	Actively restore electrical conduction Recruit endothelial cells for revascularization	(85)
Optoelectronically active scaffolds	Micro-solar cells blended with Gel-MA	Modulate cardiomyocyte beating frequency via pulsed light Synchronize with the host myocardium Prevent arrhythmogenesis	(85)

Gel-MA, methacrylated gelatin; PVDF, polyvinylidene fluoride; VMC, viral myocarditis.

#### 5.2.3 Plant-derived exosome-like nanovesicles: natural anti-inflammatory vehicles

Compared with exosomes derived from traditional mammalian cells, PELNs have gradually become a research focus in the field of nanomedicine due to their unique cross-species biological regulatory capabilities (114). PELNs are widely present in edible plant tissues and offer advantages such as inherent low immunogenicity, abundant sources, low extraction costs and ease of large-scale production, thereby overcoming, to some extent, the inherent limitations of stem cell-derived exosomes in terms of ethical concerns, batch consistency and yield (115, 116).

A growing body of research indicates that PELNs are not merely inert nanocarriers, but are rich in various bioactive lipids, small RNA molecules and metabolites, and can exert functional regulatory effects between different species (117). For example, PELNs derived from ginger (*Zingiber officinale*) and grapefruit (*Citrus paradisi*) have been shown to be naturally enriched with specific phospholipid components and microRNAs; upon uptake by host cells, these molecules can directly participate in the regulation of inflammation-related signalling pathways (118, 119). Mechanistically, plant-derived exosome-like nanovesicles (PELNs) exert targeted immunomodulatory effects by directly interacting with innate immune populations, most notably macrophages. Ginger-derived exosome-like nanoparticles are internalized by macrophages, where they actively suppress the assembly of the NLRP3 inflammasome. This intracellular blockade interrupts the inflammatory cascade at a proximal node, significantly reducing the maturation and subsequent release of key pro-inflammatory cytokines, including IL-1β and IL-18 (120). Similarly, grapefruit-derived nanovesicles exploit their inherent structural stability to efficiently cross physiological barriers, functioning as highly biocompatible carriers. By delivering targeted anti-inflammatory agents directly to the inflamed myocardium, these nanovesicles effectively attenuate local oxidative stress and mitigate cardiomyocyte apoptosis (121). This precise delivery system ensures robust microenvironmental regulation while entirely circumventing systemic toxicity.

At the molecular level, the anti-inflammatory effects of plant-derived PELNs are primarily manifested through the precise inhibition of the inflammasome signalling pathway (120, 122). This mechanism of action enables PELNs to achieve ‘upstream inhibition’ at the early stages of the inflammatory cascade, thereby effectively limiting inflammatory amplification and immune-mediated tissue damage (115).

Within the immunopathological context of VMC, the NLRP3 inflammasome is considered a key hub driving cytokine storms, inflammatory cell death and the deterioration of myocardial function (123). Consequently, the innate inflammasome-inhibitory properties exhibited by PELNs make them a highly attractive immunomodulatory vehicle, offering the potential to finely regulate the local inflammatory microenvironment of the myocardium without inducing systemic immunosuppression (124).

PELNs represent an alternative to conventional engineered nanomaterials; their combined advantages in terms of biosafety, immunotolerance and production feasibility offer new avenues for the targeted treatment of VMC and related inflammatory heart diseases in the future, whilst also laying a crucial foundation for the transition of nanomedicine from the laboratory to clinical application (**Table 4**) (125).

**Table 4:** Therapeutic mechanisms of exosomes and exosome-like nanoparticles in myocardial injury.

Biomaterial system	Bioactive cue	Significant findings	References
Ginger-derived exosome-like nanoparticles	Specific phospholipid components and microRNAs	• Directly target macrophages • Suppress NLRP3 inflammasome assembly • Significantly reduce IL-1β and IL-18 release	(118, 120, 122)
Grapefruit-derived nanovesicles	Anti-inflammatory agents	• Act as biocompatible carriers to cross physiological barriers • Deliver therapeutic agents directly to the inflamed myocardium • Attenuate local oxidative stress and cardiomyocyte apoptosis without systemic toxicity	(119, 121)
HA hydrogels or gelatin microneedle patches	Mesenchymal stem cell (MSC)-derived exosomes	• Release bioactive factors via lipid nanovesicles • Attenuate adverse cardiac remodeling • Reduce apoptosis and enhance proliferation	(85)
Fibrin hydrogels	Macrovascular endothelial cell (EC)-derived exosomes	• Deliver uniquely immunomodulatory miRNAs • Alleviate virus-mediated cardiac injury by downregulating the TLR-NF-κB axis • Enhance cardiomyocyte contractility	(85)

EC, endothelial cell; HA, hyaluronic acid; IL-1β, interleukin-1β; IL-18, interleukin-18; miRNAs, microRNAs; MSC, mesenchymal stem cell; NF-κB, nuclear factor kappa B; NLRP3, NLR family pyrin domain containing 3; TLR, Toll-like receptor.

#### 5.2.4 Biomimetic nanoparticle delivery systems: the ‘decoy’ strategy of using poison to counter poison

To further enhance the targeting efficiency of drugs in VMC and reduce systemic side effects, a biomimetic nanodelivery system based on cell membrane camouflage has been developed (126). This strategy utilises natural cell membranes to encapsulate a drug-loaded nanocore, resulting in behaviour within the body that more closely resembles that of endogenous cells or cell debris (127). Compared with conventional nanocarriers, biomimetic nanoparticles based on cell membranes demonstrate significant advantages in terms of immune evasion, circulatory stability and the ability to accumulate at the site of the lesion (128).

Currently, commonly used membrane sources include macrophage membranes, platelet membranes and erythrocyte membranes. By retaining the intact profile of transmembrane proteins and glycoproteins on the membrane surface, these biomimetic nanoparticles are able to effectively evade recognition by the immune system and rapid clearance by the reticuloendothelial system (RES), thereby significantly prolonging their circulation half-life in the blood (129, 130). This effect provides the necessary time window for drugs to achieve adequate distribution and accumulation at the site of action within the body (131).

More importantly, the cell membrane-mimicking strategy is not merely a passive means of immune evasion; it also endows nanoparticles with the ability to actively target inflammatory tissues. For example, nanoparticles coated with macrophage membranes can retain inflammatory chemotactic receptors or adhesion molecules such as CCR2 and CXCR2, thereby following the chemokine gradient highly expressed at the site of inflammation to actively migrate and accumulate in areas of the VMC myocardium prone to inflammation (132, 133). As a concrete application of this strategy, macrophage membrane-camouflaged nanoparticles displaying functional surface receptors like CCR2 have been shown to actively home to inflamed myocardial sites, neutralize pro-inflammatory cytokines, and enable the highly targeted delivery of encapsulated drugs (133).This property enables them to exhibit immune cell-like ‘homing’ behaviour within complex inflammatory microenvironments, significantly enhancing local drug delivery efficiency (134). While macrophage membranes excel at chemokine-driven homing, platelet and red blood cell (RBC) membranes offer distinct, complementary advantages tailored to different pathological facets of VMC. Platelet membrane-coated nanoparticles capitalize on inherent thrombo-inflammatory targeting. Driven by a natural affinity for damaged endothelium, these constructs selectively anchor to injured myocardial microvasculature (135, 136). Besides, platelet biomimetics can also sequester activated immune cells and modulate local immunothrombosis, directly attenuating endothelial inflammation (137). Conversely, RBC membrane-camouflaged nanoparticles are optimized for prolonged systemic circulation and broad-spectrum detoxification. By leveraging the dense surface expression of CD47 glycoproteins to engage the CD47-SIRPα signaling axis, erythrocyte membrane-camouflaged nanoparticles effectively evade phagocytic clearance by the RES (138, 139). This prolonged circulatory half-life facilitates their utility as broad-spectrum biomimetic decoys, enabling the systemic sequestration and neutralization of viral particles, cytolytic toxins, and pathogenic autoantibodies prior to the exacerbation of myocardial injury (138).

Furthermore, biomimetic nanoparticles modelled on cell membranes can adsorb free pro-inflammatory cytokines (such as TNF-α and IL-1β) or viral particles via receptors or binding sites naturally present on their surfaces, thereby partially neutralising pathogenic signals prior to drug release (140, 141). Beyond this passive sequestration, these biomimetic platforms actively orchestrate localized immune reprogramming. By competitively intercepting these extracellular cytokines and DAMPs, membrane-camouflaged nanoparticles prevent endogenous receptor activation, effectively blunting the hyperactivation of infiltrating innate immune cells and cytotoxic T lymphocytes (142). To maximize this decoy effect, advanced hybrid nanoparticles coated with fused T-lymphocyte and macrophage membranes have been specifically tailored for autoimmune myocarditis. This hybrid platform acts as a dual-functional immune decoy that sequesters pathological cytokines and delivers gene-silencing therapeutics, thereby dramatically suppressing cardiomyocyte pyroptosis, myocardial necrosis, and fibrotic remodeling *in vivo* (143).This strategy not only reduces the effective concentration of inflammatory mediators in the circulation but also creates a more favourable immunological microenvironment for targeted drug delivery (144).

In VMC—a disease characterised by inflammation amplification and immune-mediated damage—cell membrane-mimetic nanodelivery systems thus possess dual therapeutic potential: they can serve as a precision drug delivery platform, efficiently delivering anti-inflammatory or immunomodulatory molecules to damaged cardiac muscle; and they can directly attenuate inflammation and viral activity through a decoy effect (143, 145).This integrated strategy, combining ‘biological camouflage, inflammation targeting and signal neutralisation’, represents a significant shift in nanomedicine from passive carriers to active immunomodulatory tools, offering a highly promising engineering solution for the precise intervention in VMC (**Table 5**) (146).

**Table 5:** Applications of cell membrane-camouflaged nanoparticles as immune decoys and targeted delivery vehicles.

Biomaterial system	Bioactive cue	Significant findings	References
Biomimetic nanoparticles	Cell membranes (e.g., macrophage, platelet, and erythrocyte membranes)	Retain the intact profile of transmembrane proteins and glycoproteins Effectively evade immune recognition and rapid clearance by the reticuloendothelial system (RES) Significantly prolong circulation half-life	(126–131)
Membrane-camouflaged nanoparticles	Macrophage membranes (retaining CCR2, CXCR2 receptors)	Actively home to inflamed myocardial sites along chemokine gradients Neutralize pro-inflammatory cytokines Enable highly targeted delivery of encapsulated drugs	(132–134)
Hybrid nanoparticles	Fused T-lymphocyte and macrophage membranes	Act as a dual-functional immune decoy to adsorb free pro-inflammatory cytokines or viral particles Deliver gene-silencing therapeutics Dramatically suppress cardiomyocyte pyroptosis, myocardial necrosis, and fibrotic remodeling *in vivo*	(140, 141, 143–146)

CCR2, C-C chemokine receptor type 2; CXCR2, C-X-C motif chemokine receptor 2; RES, reticuloendothelial system.

### 5.3 Cell therapy and the modernisation of traditional Chinese medicine

With advances in regenerative cell therapy and the modernisation of TCM at the molecular level, the immunomodulatory strategies of VMC and DCM are becoming increasingly diverse.

#### 5.3.1 The dominant role of paracrine signalling in MSC-based therapy

MSCs have long been regarded as a promising therapeutic strategy for VMC and its subsequent myocardial lesions, owing to their potential role in tissue repair and immune modulation (147). Early research primarily focused on the possibility of MSCs differentiating into cardiomyocytes and directly integrating into damaged tissue; however, as *in vivo* tracing and functional studies have progressed, this hypothesis has gradually been revised (148). The current mainstream scientific consensus holds that the therapeutic efficacy of MSCs in cardiac disease does not primarily stem from their long-term survival or structural replacement, but rather relies on their potent paracrine effects (149).

The paracrine function of MSCs is primarily mediated by their secretome, which comprises a variety of soluble cytokines, growth factors and extracellular vesicles (EVs), with exosomes considered to be the key functional carriers (150, 151). MSC-derived exosomes, acting as natural nanoscale delivery systems, are capable of carrying various bioactive molecules such as microRNAs, proteins and lipids, and are efficiently taken up by cardiomyocytes, fibroblasts and immune cells (152). By modulating the transcription and signalling pathways of recipient cells, these exosomes exert a sustained and finely tuned regulatory effect within the local microenvironment.

In the context of VMC-induced immunopathology, MSC-derived exosomes exhibit significant anti-inflammatory and cardioprotective effects. On the one hand, they can inhibit the activation of endogenous apoptotic pathways in cardiomyocytes by delivering anti-apoptotic microRNAs (such as miR-21 and miR-146a), thereby reducing inflammation-related cardiomyocyte loss (153, 154). On the other hand, MSC-derived exosomes are capable of remodelling the immune microenvironment, significantly influencing the functional state of innate immune cells. Several studies have demonstrated that these exosomes can induce the polarisation of macrophages from the pro-inflammatory M1 phenotype to the tissue-repairing M2 phenotype by regulating signalling pathways such as STAT3 and NF-κB, thereby promoting the resolution of inflammation and myocardial repair (155, 156).

It is worth noting that this ‘cell-free’ therapeutic strategy, centred on paracrine mechanisms, offers significant advantages in terms of safety and controllability. Compared with live cell transplantation, MSC-derived exosomes avoid cell-related uncertainties, including oncogenicity, immune rejection and low retention rates *in vivo* (157). Furthermore, exosomes offer greater feasibility in terms of preparation, storage and dose standardisation, providing a practical pathway for large-scale production and clinical translation (158).

Consequently, MSC-based therapy is gradually shifting from a ‘cell replacement’ approach to one of ‘paracrine immune reprogramming’; this shift not only deepens our understanding of the mechanisms of action of MSCs, but also provides a safer, more stable and more engineering-friendly intervention pathway for the precision treatment of VMC and inflammatory cardiomyopathy (159).

#### 5.3.2 Synergizing TCM molecular precision with smart biomaterial delivery

Driven by systems immunology and molecular pharmacology, traditional Chinese medicine (TCM) is transitioning from empirical compound formulations to the precise application of bioactive monomers (160). Although bioactive TCM monomers—such as puerarin, astragaloside IV and berberine—confer cardioprotection in VMC via antiviral, antioxidant, and anti-inflammatory pathways, their clinical translation is hindered by poor aqueous solubility, low bioavailability, and a lack of cardiac-specific distribution (161–164). Integrating these structurally defined compounds with nanodelivery systems has emerged as a crucial strategy to enhance their stability and myocardial targeting.Consequently, this technological integration not only enhances the scientific verifiability of traditional therapies but also enables their incorporation into mechanism-driven frameworks for precision immune regulation, thereby unlocking new therapeutic potential for complex immune-mediated diseases such as VMC (165).

Astragaloside IV (AS-IV) is a prime example of this modernisation process. AS-IV is a major active saponin component extracted from the traditional Chinese medicinal plant Astragalus (*Astragalus membranaceus*) and has been demonstrated to possess multifaceted cardiovascular protective effects, including anti-inflammatory, antioxidant and anti-fibrotic properties (166). In studies related to VMC, the immunomodulatory effects of AS-IV have increasingly been attributed to its precise intervention in specific signalling pathways, rather than non-specific, broad-spectrum inhibition (167).

Mechanistic studies have shown that AS-IV exerts significant immunoregulatory effects by targeting the Notch signalling pathway (168). Notch signalling plays a pivotal role in T-cell fate determination; its abnormal, sustained activation drives the differentiation of naïve CD4^+^ T cells into pro-inflammatory Th17 cells, whilst simultaneously inhibiting the generation and stability of regulatory T cells (Tregs) (169, 170). In VMC and its chronic phase, this imbalance in the Th17/Treg axis is considered a major immunological basis for the maintenance of systemic inflammation and the promotion of myocardial fibrosis.

By inhibiting the abnormal activation of the Notch signalling pathway, AS-IV effectively corrects the imbalance in the Th17/Treg ratio, reduces the production of pro-inflammatory cytokines such as IL-17, and enhances Treg-mediated immune tolerance (171). In animal models, this immune remodelling effect is closely associated with reduced myocardial inflammation, decreased collagen deposition, and improved ventricular remodelling (172). To maximize this precise therapeutic potential, recent bioengineering approaches have focused on encapsulating AS-IV within biomimetic nanocarriers or stimuli-responsive hydrogels (173, 174). This integration protects the therapeutic monomer from premature degradation and facilitates localized, sustained release driven by the pathological VMC microenvironment. Consequently, high concentrations of AS-IV are maintained precisely within immune cell-rich infiltrates, ensuring the targeted modulation of the Notch/Th17-Treg axis while circumventing off-target systemic effects.

This synergy between TCM molecular pharmacology and advanced biomaterials represents a highly promising avenue for precision cardiac immunotherapy. Particularly in VMC—a pathology involving complex, multi-layered regulation of innate immunity, adaptive immunity, and fibrotic remodeling—integrating these immunologically precise natural molecules with engineered delivery platforms serves as a critical complement to existing anti-inflammatory regimens, thereby expanding the current therapeutic armamentarium (175).

## 6 Conclusions and future prospects

The progression from VMC to DCM is not the result of a single event, but rather a continuous pathological process driven by the interaction between viral pathogenicity, the host immune response, and metabolic and structural remodelling (176). The immune system plays a dual role in this process: early activation is necessary for viral clearance, but persistent or dysregulated immune responses can lead to inflammatory cell death, fibrosis, and ultimately cardiac dysfunction (177, 178). Based on current evidence, traditional systemic therapies, such as broad-spectrum immunosuppressants, face clinical limitations due to a lack of spatiotemporal specificity, as they often compromise essential antiviral defenses when suppressing myocardial inflammation (179).Therefore, the application of smart biomaterials and nanomedicine, including functionalized cardiac patches and biomimetic nanoparticles, provides a targeted therapeutic alternative (180). These platforms allow for specific modulation of the local cardiac immune microenvironment, facilitating targeted intervention without the widespread off-target effects associated with systemic immunosuppression (179).

### 6.1 Towards precise subtyping

Future clinical management should move towards precision phenotyping based on detailed immune profiling (181). For example, integrating single-cell RNA sequencing and spatial transcriptomics could help clinicians classify patients into “viral-dominant” or “immune-dominant” phenotypes (182–185). This specific diagnostic approach is important to prevent the adverse effects of administering immunosuppressive therapies to patients with active viral replication (186).

### 6.2 A new era in combination therapy

Future therapeutic regimens are expected to combine precision immunomodulators with local delivery systems (187). For instance, biologic agents targeting specific inflammatory pathways, such as the IL-1β inhibitor canakinumab or NLRP3 inflammasome inhibitors, could be encapsulated within macrophage membrane-camouflaged nanoparticles (188, 189).This combination would enable the drug to actively target inflamed myocardial sites via surface receptors like CCR2, acting as a decoy to neutralize circulating cytokines while delivering the medication directly to the injured tissue (190).

### 6.3 Overcoming conversion barriers

Moving these platforms from bench to bedside requires addressing biocompatibility and manufacturing challenges (191). For example, in the development of plant-derived exosome-like nanovesicles (PELNs) from ginger or grapefruit, researchers need to establish standardized Good Manufacturing Practice (GMP) protocols to ensure the reproducibility of their bioactive lipid and microRNA components (192, 193). Additionally, for long-term implants such as piezoelectric cardiac patches, further *in vivo* studies are necessary to evaluate whether their degradation products might induce chronic foreign body reactions or trigger VMC-related arrhythmias (194, 195).

## 7 Conclusion

In summary, integrating immunology and materials science in VMC research is essential for addressing the clinical challenges of the VMC-DCM transition (196). By regulating the spatiotemporal aspects of the immune response through engineered biomaterials, it may become possible to better predict, stratify, and proactively prevent heart failure following myocarditis (197).

## References

[1] Ricci JC Farahani NA Davis CJ Ritter KG Parrow LM Tomerlin PI . A comparative review of myocarditis in pediatrics versus adults: pathogenesis, diagnosis, and management. Front Immunol. (2025) 16:1601307. doi: 10.3389/fimmu.2025.1601307 41080571 PMC12511060

[2] Dawood I Alhussein ST Wadi WYA Abdalgadir RAY Mohammed SSI Ahmed EHM . Viral myocarditis in pediatrics: A review of current diagnostic methods and future directions. Ann Pediatr Cardiol. (2025) 18:42–8. doi: 10.4103/apc.apc_236_24 40814331 PMC12348764

[3] Nappi F . Myocarditis and inflammatory cardiomyopathy in dilated heart failure. Viruses. (2025) 17:484. doi: 10.3390/v17040484 40284927 PMC12031395

[4] Wang J Lu W Zhang J Du Y Fang M Zhang A . Loss of TRIM29 mitigates viral myocarditis by attenuating PERK-driven ER stress response in male mice. Nat Commun. (2024) 15:3481. doi: 10.1038/s41467-024-44745-x 38664417 PMC11045800

[5] Zhang Y Zhou X Chen S Sun X Zhou C . Immune mechanisms of group B coxsackievirus induced viral myocarditis. Virulence. (2023) 14:2180951. doi: 10.1080/21505594.2023.2180951 36827455 PMC9980623

[6] Xu J Zhou Z Zheng Y Yang S Huang K Li H . Roles of inflammasomes in viral myocarditis. Front Cell Infect Microbiol. (2023) 13:1149911. doi: 10.3389/fcimb.2023.1149911 37256114 PMC10225676

[7] Imanaka-Yoshida K . Inflammation in myocardial disease: From myocarditis to dilated cardiomyopathy. Pathol Int. (2020) 70:1–11. doi: 10.1111/pin.12868 31691489

[8] Golino M Harding D Del Buono MG Fanti S Mohiddin S Toldo S . Innate and adaptive immunity in acute myocarditis. Int J Cardiol. (2024) 404:131901. doi: 10.1016/j.ijcard.2024.131901 38403204 PMC11450758

[9] Wei X Fang Y Hu H . Glucocorticoid and immunoglobulin to treat viral fulminant myocarditis. Eur Heart J. (2020) 41:2122. doi: 10.1093/eurheartj/ehaa357 32338741 PMC7279514

[10] Sarzani R Spannella F Giulietti F Di Pentima C Giordano P Giacometti A . Possible harm from glucocorticoid drugs misuse in the early phase of SARS-CoV-2 infection: a narrative review of the evidence. Intern Emerg Med. (2022) 17:329–38. doi: 10.1007/s11739-021-02860-3 34718937 PMC8557262

[11] Carty M Guy C Bowie AG . Detection of viral infections by innate immunity. Biochem Pharmacol. (2021) 183:114316. doi: 10.1016/j.bcp.2020.114316 33152343

[12] Gorbea C Makar KA Pauschinger M Pratt G Bersola JL Varela J . A role for Toll-like receptor 3 variants in host susceptibility to enteroviral myocarditis and dilated cardiomyopathy. J Biol Chem. (2010) 285:23208–23. doi: 10.1074/jbc.m109.047464 20472559 PMC2906314

[13] Zhang W Lavine KJ Epelman S Evans SA Weinheimer CJ Barger PM . Necrotic myocardial cells release damage-associated molecular patterns that provoke fibroblast activation *in vitro* and trigger myocardial inflammation and fibrosis *in vivo*. J Am Heart Assoc. (2015) 4:e001993. doi: 10.1161/jaha.115.001993 26037082 PMC4599537

[14] Ren W Zhao L Sun Y Wang X Shi X . HMGB1 and Toll-like receptors: potential therapeutic targets in autoimmune diseases. Mol Med. (2023) 29:117. doi: 10.1186/s10020-023-00717-3 37667233 PMC10478470

[15] Decout A Katz JD Venkatraman S Ablasser A . The cGAS-STING pathway as a therapeutic target in inflammatory diseases. Nat Rev Immunol. (2021) 21:548–69. doi: 10.1038/s41577-021-00524-z 33833439 PMC8029610

[16] Wang W Gao Y Lee HK Yu AC Kipp M Kaddatz H . The cGAS/STING pathway: Friend or foe in regulating cardiomyopathy. Cells. (2025) 14:778. doi: 10.20944/preprints202504.2184.v1 PMC12153880 40497954

[17] Kelly B O'Neill LA . Metabolic reprogramming in macrophages and dendritic cells in innate immunity. Cell Res. (2015) 25:771–84. doi: 10.1038/cr.2015.68 26045163 PMC4493277

[18] Luo L Zhuang X Fu L Dong Z Yi S Wang K . The role of the interplay between macrophage glycolytic reprogramming and NLRP3 inflammasome activation in acute lung injury/acute respiratory distress syndrome. Clin Transl Med. (2024) 14:e70098. doi: 10.1002/ctm2.70098 39623879 PMC11612265

[19] Gong N Wang W Fu Y Zheng X Guo X Chen Y . The crucial role of metabolic reprogramming in driving macrophage conversion in kidney disease. Cell Mol Biol Lett. (2025) 30:72. doi: 10.1186/s11658-025-00746-2 40524149 PMC12172235

[20] Zhuang L Zong X Yang Q Fan Q Tao R . Interleukin-34-NF-κB signaling aggravates myocardial ischemic/reperfusion injury by facilitating macrophage recruitment and polarization. EBioMedicine. (2023) 95:104744. doi: 10.1016/j.ebiom.2023.104744 37556943 PMC10433018

[21] Feng G Bajpai G Ma P Koenig A Bredemeyer A Lokshina I . CCL17 aggravates myocardial injury by suppressing recruitment of regulatory T cells. Circulation. (2022) 145:765–82. doi: 10.1161/circulationaha.121.055888 35113652 PMC8957788

[22] Ichimura S Misaka T Ogawara R Tomita Y Anzai F Sato Y . Neutrophil extracellular traps in myocardial tissue drive cardiac dysfunction and adverse outcomes in patients with heart failure with dilated cardiomyopathy. Circ Heart Fail. (2024) 17:e011057. doi: 10.1016/s0735-1097(24)02357-x 38847093

[23] Silvestre-Roig C Braster Q Wichapong K Lee EY Teulon JM Berrebeh N . Externalized histone H4 orchestrates chronic inflammation by inducing lytic cell death. Nature. (2019) 569:236–40. doi: 10.1038/s41586-019-1167-6 31043745 PMC6716525

[24] Martín P Sánchez-Madrid F . T cells in cardiac health and disease. J Clin Invest. (2025) 135:e185218. doi: 10.1172/JCI185218 PMC11735099 39817455

[25] Baldeviano GC Barin JG Talor MV Srinivasan S Bedja D Zheng D . Interleukin-17A is dispensable for myocarditis but essential for the progression to dilated cardiomyopathy. Circ Res. (2010) 106:1646–55. doi: 10.1161/circresaha.109.213157 20378858

[26] Bansal SS Ismahil MA Goel M Zhou G Rokosh G Hamid T . Dysfunctional and proinflammatory regulatory T-lymphocytes are essential for adverse cardiac remodeling in ischemic cardiomyopathy. Circulation. (2019) 139:206–21. doi: 10.1161/circulationaha.118.036065 30586716 PMC6322956

[27] Vdovenko D Eriksson U . Regulatory role of CD4(+) T cells in myocarditis. J Immunol Res. (2018) 2018:4396351. doi: 10.1155/2018/4396351 PMC6032977 30035131

[28] Hay ZLZ Slansky JE . Granzymes: The molecular executors of immune-mediated cytotoxicity. Int J Mol Sci. (2022) 23:1833. doi: 10.3390/ijms23031833 35163755 PMC8836949

[29] Voskoboinik I Whisstock JC Trapani JA . Perforin and granzymes: function, dysfunction and human pathology. Nat Rev Immunol. (2015) 15:388–400. doi: 10.1038/nri3839 25998963

[30] Lasrado N Borcherding N Arumugam R Starr TK Reddy J . Dissecting the cellular landscape and transcriptome network in viral myocarditis by single-cell RNA sequencing. iScience. (2022) 25:103865. doi: 10.1016/j.isci.2022.103865 35243228 PMC8861636

[31] Lasrado N Reddy J . An overview of the immune mechanisms of viral myocarditis. Rev Med Virol. (2020) 30:1–14. doi: 10.1002/rmv.2131 32720461

[32] Tschöpe C Ammirati E Bozkurt B Caforio ALP Cooper LT Felix SB . Myocarditis and inflammatory cardiomyopathy: current evidence and future directions. Nat Rev Cardiol. (2021) 18:169–93. doi: 10.1038/s41569-020-00435-x PMC7548534 33046850

[33] Jane-wit D Altuntas CZ Johnson JM Yong S Wickley PJ Clark P . Beta 1-adrenergic receptor autoantibodies mediate dilated cardiomyopathy by agonistically inducing cardiomyocyte apoptosis. Circulation. (2007) 116:399–410. doi: 10.1161/circulationaha.106.683193 17620508

[34] Sharma BR Choudhury SM Abdelaal HM Wang Y Kanneganti TD . Innate immune sensor NLRP3 drives PANoptosome formation and PANoptosis. J Immunol. (2025) 214:1236–46. doi: 10.1093/jimmun/vkaf042 40249072 PMC12207079

[35] Broz P . Pyroptosis: molecular mechanisms and roles in disease. Cell Res. (2025) 35:334–44. doi: 10.1038/s41422-025-01107-6 40181184 PMC12012027

[36] Qin X Zhou Y Jia C Chao Z Qin H Liang J . Caspase-1-mediated extracellular vesicles derived from pyroptotic alveolar macrophages promote inflammation in acute lung injury. Int J Biol Sci. (2022) 18:1521–38. doi: 10.7150/ijbs.66477 35280692 PMC8898368

[37] Park TJ Park JH Lee GS Lee JY Shin JH Kim MW . Quantitative proteomic analyses reveal that GPX4 downregulation during myocardial infarction contributes to ferroptosis in cardiomyocytes. Cell Death Dis. (2019) 10:835. doi: 10.1038/s41419-019-2061-8 31685805 PMC6828761

[38] Jiang W Adamo L Lim K Matkovich SJ Evans S Rocha-Resende C . Necrotic cardiac myocytes skew macrophage polarization towards a classically activated phenotype. PloS One. (2023) 18:e0282921. doi: 10.1371/journal.pone.0282921 36996254 PMC10062615

[39] Xu S Chaudhary O Rodríguez-Morales P Sun X Chen D Zappasodi R . Uptake of oxidized lipids by the scavenger receptor CD36 promotes lipid peroxidation and dysfunction in CD8(+) T cells in tumors. Immunity. (2021) 54:1561–1577.e7. doi: 10.1016/j.immuni.2021.05.003 34102100 PMC9273026

[40] Newman LE Weiser Novak S Rojas GR Tadepalle N Schiavon CR Grotjahn DA . Mitochondrial DNA replication stress triggers a pro-inflammatory endosomal pathway of nucleoid disposal. Nat Cell Biol. (2024) 26:194–206. doi: 10.1038/s41556-023-01343-1 38332353 PMC11026068

[41] Yan M Li Y Luo Q Zeng W Shao X Li L . Mitochondrial damage and activation of the cytosolic DNA sensor cGAS-STING pathway lead to cardiac pyroptosis and hypertrophy in diabetic cardiomyopathy mice. Cell Death Discov. (2022) 8:258. doi: 10.1038/s41420-022-01046-w 35538059 PMC9091247

[42] Han Y Xu X Tang C Gao P Chen X Xiong X . Reactive oxygen species promote tubular injury in diabetic nephropathy: The role of the mitochondrial ros-txnip-nlrp3 biological axis. Redox Biol. (2018) 16:32–46. doi: 10.1016/j.redox.2018.02.013 29475133 PMC5842313

[43] Sorescu AM Coman OA Raoul-Vasile L Duica G Alin N Cinteză EE . Intravenous immunoglobulin efficacy and safety in paediatric patients diagnosed with acute myocarditis. J Clin Med. (2025) 14:7835. doi: 10.3390/jcm14217835 41227228 PMC12608166

[44] Yen CY Hung MC Wong YC Chang CY Lai CC Wu KG . Role of intravenous immunoglobulin therapy in the survival rate of pediatric patients with acute myocarditis: A systematic review and meta-analysis. Sci Rep. (2019) 9:10459. doi: 10.1038/s41598-019-46888-0 31320679 PMC6639391

[45] Spirig R Campbell IK Koernig S Chen CG Lewis BJB Butcher R . rIgG1 Fc hexamer inhibits antibody-mediated autoimmune disease via effects on complement and FcγRs. J Immunol. (2018) 200:2542–53. doi: 10.4049/jimmunol.1701171 29531170 PMC5890536

[46] Sinkovits G Schnur J Hurler L Kiszel P Prohászka ZZ Sík P . Evidence, detailed characterization and clinical context of complement activation in acute multisystem inflammatory syndrome in children. Sci Rep. (2022) 12:19759. doi: 10.1038/s41598-022-23806-5 36396679 PMC9670087

[47] Robinson J Hartling L Vandermeer B Sebastianski M Klassen TP . Intravenous immunoglobulin for presumed viral myocarditis in children and adults. Cochrane Database Syst Rev. (2020) 8:Cd004370. doi: 10.1002/14651858.cd004370.pub3 32835416 PMC8210245

[48] Hazebroek MR Henkens M Raafs AG Verdonschot JAJ Merken JJ Dennert RM . Intravenous immunoglobulin therapy in adult patients with idiopathic chronic cardiomyopathy and cardiac parvovirus B19 persistence: a prospective, double-blind, randomized, placebo-controlled clinical trial. Eur J Heart Fail. (2021) 23:302–9. doi: 10.1002/ejhf.2082 33347677 PMC8048650

[49] Bazzone LE Zhu J King M Liu G Guo Z MacKay CR . ADAM9 promotes type I interferon-mediated innate immunity during encephalomyocarditis virus infection. Nat Commun. (2024) 15:4153. doi: 10.1038/s41467-024-48524-6 38755212 PMC11098812

[50] Wang W Xu L Su J Peppelenbosch MP Pan Q . Transcriptional regulation of antiviral interferon-stimulated genes. Trends Microbiol. (2017) 25:573–84. doi: 10.1016/j.tim.2017.01.001 28139375 PMC7127685

[51] Schultheiss HP Piper C Sowade O Waagstein F Kapp JF Wegscheider K . Betaferon in chronic viral cardiomyopathy (BICC) trial: Effects of interferon-β treatment in patients with chronic viral cardiomyopathy. Clin Res Cardiol. (2016) 105:763–73. doi: 10.1007/s00392-016-0986-9 27112783

[52] Wang YX da Cunha V Vincelette J White K Velichko S Xu Y . Antiviral and myocyte protective effects of murine interferon-beta and -{alpha}2 in coxsackievirus B3-induced myocarditis and epicarditis in Balb/c mice. Am J Physiol Heart Circ Physiol. (2007) 293:H69–76. doi: 10.1152/ajpheart.00154.2007 17434974

[53] King KR Aguirre AD Ye YX Sun Y Roh JD Ng RP Jr. . IRF3 and type I interferons fuel a fatal response to myocardial infarction. Nat Med. (2017) 23:1481–7. doi: 10.1038/nm.4428 29106401 PMC6477926

[54] Kühl U Pauschinger M Schwimmbeck PL Seeberg B Lober C Noutsias M . Interferon-beta treatment eliminates cardiotropic viruses and improves left ventricular function in patients with myocardial persistence of viral genomes and left ventricular dysfunction. Circulation. (2003) 107:2793–8. doi: 10.1161/01.CIR.0000072766.67150.51 12771005

[55] Li H Chen X Wang JJ Shen J Abuduwufuer K Zhang Z . Spatiotemporal transcriptomics elucidates the pathogenesis of fulminant viral myocarditis. Signal Transduct Target Ther. (2025) 10:59. doi: 10.1038/s41392-025-02143-9 39924580 PMC11808084

[56] Przytuła N Podolec J Przewłocki T Podolec P Kabłak-Ziembicka A . Inflammasomes as potential therapeutic targets to prevent chronic active viral myocarditis-translating basic science into clinical practice. Int J Mol Sci. (2025) 26:11003. doi: 10.3390/ijms262211003 PMC12652514 41303485

[57] Libby P . Targeting inflammatory pathways in cardiovascular disease: The inflammasome, interleukin-1, interleukin-6 and beyond. Cells. (2021) 10:951. doi: 10.3390/cells10040951 33924019 PMC8073599

[58] Ridker PM Thuren T Zalewski A Libby P . Interleukin-1β inhibition and the prevention of recurrent cardiovascular events: rationale and design of the Canakinumab Anti-inflammatory Thrombosis Outcomes Study (CANTOS). Am Heart J. (2011) 162:597–605. doi: 10.1016/j.ahj.2011.06.012 21982649

[59] Ridker PM MacFadyen JG Thuren T Libby P . Residual inflammatory risk associated with interleukin-18 and interleukin-6 after successful interleukin-1β inhibition with canakinumab: further rationale for the development of targeted anti-cytokine therapies for the treatment of atherothrombosis. Eur Heart J. (2020) 41:2153–63. doi: 10.1093/eurheartj/ehz542 31504417

[60] Huang J Kuang W Zhou Z . IL-1 signaling pathway, an important target for inflammation surrounding in myocardial infarction. Inflammopharmacology. (2024) 32:2235–52. doi: 10.1007/s10787-024-01481-4 38676853

[61] Saha A Sharma AR Bhattacharya M Sharma G Lee SS Chakraborty C . Tocilizumab: A therapeutic option for the treatment of cytokine storm syndrome in COVID-19. Arch Med Res. (2020) 51:595–7. doi: 10.1016/j.arcmed.2020.05.009 32482373 PMC7241374

[62] Wang H Tian R Gao P Wang Q Zhang L . Tocilizumab for fulminant programmed death 1 inhibitor-associated myocarditis. J Thorac Oncol. (2020) 15:e31–2. doi: 10.1002/9781119453192.ch48 32093854

[63] Alazawi W Pirmadjid N Lahiri R Bhattacharya S . Inflammatory and immune responses to surgery and their clinical impact. Ann Surg. (2016) 264:73–80. doi: 10.1097/sla.0000000000001691 27275778

[64] Li X Huang C Rai KR Xu Q . Innate immune role of IL-6 in influenza a virus pathogenesis. Front Cell Infect Microbiol. (2025) 15:1605446. doi: 10.3389/fcimb.2025.1605446 40692679 PMC12277322

[65] Haley KE Almas T Shoar S Shaikh S Azhar M Cheema FH . The role of anti-inflammatory drugs and nanoparticle-based drug delivery models in the management of ischemia-induced heart failure. BioMed Pharmacother. (2021) 142:112014. doi: 10.1016/j.biopha.2021.112014 34391184

[66] Jensen LD Marchant DJ . Emerging pharmacologic targets and treatments for myocarditis. Pharmacol Ther. (2016) 161:40–51. doi: 10.1016/j.pharmthera.2016.03.006 27009690

[67] De Luca G Campochiaro C Sartorelli S Peretto G Dagna L . Therapeutic strategies for virus-negative myocarditis: a comprehensive review. Eur J Intern Med. (2020) 77:9–17. doi: 10.1016/j.ejim.2020.04.050 32402564

[68] Yu C Qiu Y Yao F Wang C Li J . Chemically programmed hydrogels for spatiotemporal modulation of the cardiac pathological microenvironment. Adv Mater. (2024) 36:e2404264. doi: 10.1002/adma.202404264 38830198

[69] Wang C Luo H . Crosstalk between innate immunity and autophagy in viral myocarditis leading to dilated cardiomyopathy. Rev Med Virol. (2024) 34:e2586. doi: 10.1002/rmv.2586 39349889

[70] Nakkala JR Li Z Ahmad W Wang K Gao C . Immunomodulatory biomaterials and their application in therapies for chronic inflammation-related diseases. Acta Biomater. (2021) 123:1–30. doi: 10.1016/j.actbio.2021.01.025 33484912

[71] Zheng JB Li XY Zhu JM Liu C Song XT Wang B . Engineered immune-driven theranostics for clinical cardiology. Mil Med Res. (2025) 12:76. doi: 10.1186/s40779-025-00664-6 41188991 PMC12587584

[72] Chen W Wang C Liu W Zhao B Zeng Z Long F . A matrix-metalloproteinase-responsive hydrogel system for modulating the immune microenvironment in myocardial infarction. Adv Mater. (2023) 35:e2209041. doi: 10.1002/adma.202209041 36754377

[73] Pei W Zhang Y Zhu X Zhao C Li X Lü H . Multitargeted immunomodulatory therapy for viral myocarditis by engineered extracellular vesicles. ACS Nano. (2024) 18:2782–99. doi: 10.1021/acsnano.3c05847 38232382

[74] Rafique M Ali O Niazi M Zhang J Shafiq M Fang J . Advances in surface biofunctionalization and intelligent monitoring of vascular scaffolds. Res (Wash D C). (2026) 9:1182. doi: 10.34133/research.1182 41822912 PMC12976488

[75] Tan Y Nie Y ZhengWen L Zheng Z . Comparative effectiveness of myocardial patches and intramyocardial injections in treating myocardial infarction with a MitoQ/hydrogel system. J Mater Chem B. (2024) 12:5838–47. doi: 10.1039/d4tb00573b 38771306

[76] Rakshit P Giri TK Mukherjee K . Progresses and perspectives on natural polysaccharide based hydrogels for repair of infarcted myocardium. Int J Biol Macromol. (2024) 269:132213. doi: 10.1016/j.ijbiomac.2024.132213 38729464

[77] Eliwa A Abbas MK Al-Ejji M Zadeh K Aboujassoum H . Synthetic-polymer-based cardiac patches for MI-induced heart failure treatment: A review. Biomolecules. (2026) 16:580. doi: 10.3390/biom16040580 42072700 PMC13113715

[78] Liu T Hao Y Zhang Z Zhou H Peng S Zhang D . Advanced cardiac patches for the treatment of myocardial infarction. Circulation. (2024) 149:2002–20. doi: 10.1161/circulationaha.123.067097 38885303 PMC11191561

[79] Ho TC Chang CC Chan HP Chung TW Shu CW Chuang KP . Hydrogels: Properties and applications in biomedicine. Molecules. (2022) 27:2902. doi: 10.3390/molecules27092902 35566251 PMC9104731

[80] Xu Q Xiao Z Yang Q Yu T Deng X Chen N . Hydrogel-based cardiac repair and regeneration function in the treatment of myocardial infarction. Mater Today Bio. (2024) 25:100978. doi: 10.1016/j.mtbio.2024.100978 38434571 PMC10907859

[81] Rodell CB Lee ME Wang H Takebayashi S Takayama T Kawamura T . Injectable shear-thinning hydrogels for minimally invasive delivery to infarcted myocardium to limit left ventricular remodeling. Circ Cardiovasc Interv. (2016) 9:e004058. doi: 10.1161/circinterventions.116.004058 27729419 PMC5123705

[82] Li R Xiang C Li Y Nie Y . Targeting immunoregulation for cardiac regeneration. J Mol Cell Cardiol. (2023) 177:1–8. doi: 10.1016/j.yjmcc.2023.02.003 36801268

[83] Zhu D Li Z Huang K Caranasos TG Rossi JS Cheng K . Minimally invasive delivery of therapeutic agents by hydrogel injection into the pericardial cavity for cardiac repair. Nat Commun. (2021) 12:1412. doi: 10.1038/s41467-021-21682-7 33658506 PMC7930285

[84] Liu W Long L Wang Z He S Han Y Yang L . A whole-course-repair system based on stimulus-responsive multifunctional hydrogels for myocardial tissue regeneration. Small Methods. (2024) 8:e2400121. doi: 10.1002/smtd.202400121 38923800

[85] Shafiq M Majid QA Rafique M Talman V . Tissue-inducing biomaterials for cardiac tissue regeneration and repair. Tissue Eng Part A. (2026) 32:273–87. doi: 10.1177/19373341251404075 41468067

[86] Liguori TTA Liguori GR van Dongen JA Moreira LFP Harmsen MC . Bioactive decellularized cardiac extracellular matrix-based hydrogel as a sustained-release platform for human adipose tissue-derived stromal cell-secreted factors. BioMed Mater. (2021) 16:025022. doi: 10.1088/1748-605x/abcff9 33264764

[87] Chang C Yan J Yao Z Zhang C Li X Mao HQ . Effects of mesenchymal stem cell-derived paracrine signals and their delivery strategies. Adv Healthc Mater. (2021) 10:e2001689. doi: 10.1002/adhm.202001689 33433956 PMC7995150

[88] Eleuteri S Fierabracci A . Insights into the secretome of mesenchymal stem cells and its potential applications. Int J Mol Sci. (2019) 20:4597. doi: 10.3390/ijms20184597 31533317 PMC6770239

[89] Shang Q Dong Y Su Y Leslie F Sun M Wang F . Local scaffold-assisted delivery of immunotherapeutic agents for improved cancer immunotherapy. Adv Drug Delivery Rev. (2022) 185:114308. doi: 10.1016/j.addr.2022.114308 35472398

[90] Shafiq M Ali O Han SB Kim DH . Mechanobiological strategies to enhance stem cell functionality for regenerative medicine and tissue engineering. Front Cell Dev Biol. (2021) 9:747398. doi: 10.3389/fcell.2021.747398 34926444 PMC8678455

[91] Cheng Q Shafiq M Rafique M Shen L Mo X Wang K . Extracellular matrix and nitric oxide based functional coatings for vascular stents. Eng Regener. (2022) 3:149–53. doi: 10.1016/j.engreg.2022.03.00242574925

[92] Heo JS Choi Y Kim HO . Adipose-derived mesenchymal stem cells promote M2 macrophage phenotype through exosomes. Stem Cells Int. (2019) 2019:7921760. doi: 10.1155/2019/7921760 31781246 PMC6875419

[93] Kino T Khan M Mohsin S . The regulatory role of T cell responses in cardiac remodeling following myocardial infarction. Int J Mol Sci. (2020) 21:5013. doi: 10.3390/ijms21145013 32708585 PMC7404395

[94] Chaudhuri O Cooper-White J Janmey PA Mooney DJ Shenoy VB . Effects of extracellular matrix viscoelasticity on cellular behaviour. Nature. (2020) 584:535–46. doi: 10.1038/s41586-020-2612-2 32848221 PMC7676152

[95] Liu W Zhang X Jiang X Dai B Zhang L Zhu Y . Decellularized extracellular matrix materials for treatment of ischemic cardiomyopathy. Bioact Mater. (2024) 33:460–82. doi: 10.1016/j.bioactmat.2023.10.015 38076651 PMC10697850

[96] D'Amore A Yoshizumi T Luketich SK Wolf MT Gu X Cammarata M . Bi-layered polyurethane - extracellular matrix cardiac patch improves ischemic ventricular wall remodeling in a rat model. Biomaterials. (2016) 107:1–14. doi: 10.1016/j.biomaterials.2016.07.039 27579776

[97] van Putten S Shafieyan Y Hinz B . Mechanical control of cardiac myofibroblasts. J Mol Cell Cardiol. (2016) 93:133–42. doi: 10.1016/j.yjmcc.2015.11.025 26620422

[98] Cho S Rhee S Madl CM Caudal A Thomas D Kim H . Selective inhibition of stromal mechanosensing suppresses cardiac fibrosis. Nature. (2025) 642:766–75. doi: 10.1038/s41586-025-08945-9 40307543 PMC12176515

[99] Janssens K Bovendeerd PHM . Impact of cardiac patch alignment on restoring post-infarct ventricular function. Biomech Model Mechanobiol. (2024) 23:1963–76. doi: 10.1007/s10237-024-01877-9 39088120 PMC11639681

[100] Pilla JJ Blom AS Brockman DJ Ferrari VA Yuan Q Acker MA . Passive ventricular constraint to improve left ventricular function and mechanics in an ovine model of heart failure secondary to acute myocardial infarction. J Thorac Cardiovasc Surg. (2003) 126:1467–76. doi: 10.1016/s0022-5223(03)00739-6 14666021

[101] Herum KM Choppe J Kumar A Engler AJ McCulloch AD . Mechanical regulation of cardiac fibroblast profibrotic phenotypes. Mol Biol Cell. (2017) 28:1871–82. doi: 10.1091/mbc.e17-01-0014 28468977 PMC5541838

[102] El-Shafeey M Pappritz K Voss I Miteva K Alogna A Seifert M . Mitigating murine acute and chronic Coxsackievirus B3-induced myocarditis with human right atrial appendage-derived stromal cells. Stem Cells Transl Med. (2025) 14:szae103. doi: 10.1093/stcltm/szae103 40110808 PMC11923745

[103] Chen J Zhan Y Wang Y Han D Tao B Luo Z . Chitosan/silk fibroin modified nanofibrous patches with mesenchymal stem cells prevent heart remodeling post-myocardial infarction in rats. Acta Biomater. (2018) 80:154–68. doi: 10.1016/j.actbio.2018.09.013 30218777

[104] Zhou N Zhao Y Jiang J Shen L Li J Wan J . Impact of mechanical circulatory support and immunomodulation therapy on outcome of patients with fulminant myocarditis: Chinese registry of fulminant myocarditis. Signal Transduct Target Ther. (2021) 6:350. doi: 10.1038/s41392-021-00700-6 34611131 PMC8492670

[105] Xu M Su T Jin X Li Y Yao Y Liu K . Inflammation-mediated matrix remodeling of extracellular matrix-mimicking biomaterials in tissue engineering and regenerative medicine. Acta Biomater. (2022) 151:106–17. doi: 10.1016/j.actbio.2022.08.015 35970482

[106] Zheng Q Shi B Li Z Wang ZL . Recent progress on piezoelectric and triboelectric energy harvesters in biomedical systems. Adv Sci (Weinh). (2017) 4:1700029. doi: 10.1002/advs.201700029 28725529 PMC5515112

[107] Zhang Y Le Friec A Zhang Z Müller CA Du T Dong M . Electroactive biomaterials synergizing with electrostimulation for cardiac tissue regeneration and function-monitoring. Mater Today. (2023) 70:237–72. doi: 10.1016/j.mattod.2023.09.00542574925

[108] Wu H Dong H Tang Z Chen Y Liu Y Wang M . Electrical stimulation of piezoelectric BaTiO3 coated Ti6Al4V scaffolds promotes anti-inflammatory polarization of macrophages and bone repair via MAPK/JNK inhibition and OXPHOS activation. Biomaterials. (2023) 293:121990. doi: 10.1016/j.biomaterials.2022.121990 36586147

[109] Fadle Aziz MR Wlodarek L Alibhai F Wu J Li S Sun Y . A polypyrrole-polycarbonate polyurethane elastomer alleviates cardiac arrhythmias via improving bio-conductivity. Adv Healthc Mater. (2023) 12:e2203168. doi: 10.1002/adhm.202203168 36849128

[110] Huang H Wang K Liu X Wang J Suo M Liu X . Can self-powered piezoelectric materials be used to treat disc degeneration by means of electrical stimulation? Front Bioeng Biotechnol. (2024) 12:1397261. doi: 10.3389/fbioe.2024.1397261 38784767 PMC11111946

[111] Xia R Tomsits P Loy S Zhang Z Pauly V Schüttler D . Cardiac macrophages and their effects on arrhythmogenesis. Front Physiol. (2022) 13:900094. doi: 10.3389/fphys.2022.900094 35812333 PMC9257039

[112] Chen S Hsieh MH Li SH Wu J Weisel RD Chang Y . A conductive cell-delivery construct as a bioengineered patch that can improve electrical propagation and synchronize cardiomyocyte contraction for heart repair. J Control Release. (2020) 320:73–82. doi: 10.1016/j.jconrel.2020.01.027 31958479

[113] Esmaeili H Patino-Guerrero A Hasany M Ansari MO Memic A Dolatshahi-Pirouz A . Electroconductive biomaterials for cardiac tissue engineering. Acta Biomater. (2022) 139:118–40. doi: 10.1016/j.actbio.2021.08.031 34455109 PMC8935982

[114] Song Y Feng N Yu Q Li Y Meng M Yang X . Exosomes in disease therapy: Plant-derived exosome-like nanoparticles current status, challenges, and future prospects. Int J Nanomedicine. (2025) 20:10613–44. doi: 10.2147/ijn.s540094 40918944 PMC12410150

[115] Yi Q Xu Z Thakur A Zhang K Liang Q Liu Y . Current understanding of plant-derived exosome-like nanoparticles in regulating the inflammatory response and immune system microenvironment. Pharmacol Res. (2023) 190:106733. doi: 10.1016/j.phrs.2023.106733 36931541

[116] Bai C Liu J Zhang X Li Y Qin Q Song H . Research status and challenges of plant-derived exosome-like nanoparticles. BioMed Pharmacother. (2024) 174:116543. doi: 10.1016/j.biopha.2024.116543 38608523

[117] Han Y Guo X Ji Z Guo Y Ma W Du H . Colon health benefits of plant-derived exosome-like nanoparticles via modulating gut microbiota and immunity. Crit Rev Food Sci Nutr. (2025) 65:7718–38. doi: 10.1080/10408398.2025.2479066 40105379

[118] Yin L Yan L Yu Q Wang J Liu C Wang L . Characterization of the microRNA profile of ginger exosome-like nanoparticles and their anti-inflammatory effects in intestinal Caco-2 cells. J Agric Food Chem. (2022) 70:4725–34. doi: 10.1021/acs.jafc.1c07306 35261246

[119] Yi C Lu L Li Z Guo Q Ou L Wang R . Plant-derived exosome-like nanoparticles for microRNA delivery in cancer treatment. Drug Delivery Transl Res. (2025) 15:84–101. doi: 10.1007/s13346-024-01621-x 38758499

[120] Chen X Zhou Y Yu J . Exosome-like nanoparticles from ginger rhizomes inhibited NLRP3 inflammasome activation. Mol Pharm. (2019) 16:2690–9. doi: 10.1021/acs.molpharmaceut.9b00246 31038962

[121] Savcı Y Kırbaş OK Bozkurt BT Abdik EA Taşlı PN Şahin F . Grapefruit-derived extracellular vesicles as a promising cell-free therapeutic tool for wound healing. Food Funct. (2021) 12:5144–56. doi: 10.1039/d0fo02953j 33977960

[122] Lv Q Yang H Xie Y Huang X Yan Z Lv Y . Prunus mume derived extracellular vesicle-like particles alleviate experimental colitis via disrupting NEK7-NLRP3 interaction and inflammasome activation. J Nanobiotechnology. (2025) 23:532. doi: 10.1186/s12951-025-03567-9 40685348 PMC12278495

[123] Toldo S Abbate A . The role of the NLRP3 inflammasome and pyroptosis in cardiovascular diseases. Nat Rev Cardiol. (2024) 21:219–37. doi: 10.1038/s41569-023-00946-3 37923829 PMC11550901

[124] Cheng Z Lu W Shao W Zhang C She Y Song R . Advances in plant-derived vesicle like nanoparticles-based therapies for inflammatory diseases. Asian J Pharm Sci. (2025) 20:101052. doi: 10.1016/j.ajps.2025.101052 40777842 PMC12329537

[125] Zhao B Lin H Jiang X Li W Gao Y Li M . Exosome-like nanoparticles derived from fruits, vegetables, and herbs: Innovative strategies of therapeutic and drug delivery. Theranostics. (2024) 14:4598–621. doi: 10.7150/thno.97096 39239509 PMC11373634

[126] Zhang Z Xiong Y Liu S Shen L Zheng L Ding L . Nanoparticles coated with immune cell hybrid membranes for targeted delivery of Janus kinase inhibitors and synergistic treatment of autoimmune myocarditis. Acta Biomater. (2025) 205:584–600. doi: 10.1016/j.actbio.2025.09.013 40945884

[127] Fang RH Gao W Zhang L . Targeting drugs to tumours using cell membrane-coated nanoparticles. Nat Rev Clin Oncol. (2023) 20:33–48. doi: 10.1038/s41571-022-00699-x 36307534

[128] Dash P Piras AM Dash M . Cell membrane coated nanocarriers - an efficient biomimetic platform for targeted therapy. J Control Release. (2020) 327:546–70. doi: 10.1016/j.jconrel.2020.09.012 32911013

[129] Zhao X Yan C . Research progress of cell membrane biomimetic nanoparticles for tumor therapy. Nanoscale Res Lett. (2022) 17:36. doi: 10.1186/s11671-022-03673-9 35316443 PMC8941025

[130] Wang H Liu Y He R Xu D Zang J Weeranoppanant N . Cell membrane biomimetic nanoparticles for inflammation and cancer targeting in drug delivery. Biomater Sci. (2020) 8:552–68. doi: 10.1039/c9bm01392j 31769765

[131] Haroon HB Hunter AC Farhangrazi ZS Moghimi SM . A brief history of long circulating nanoparticles. Adv Drug Delivery Rev. (2022) 188:114396. doi: 10.1016/j.addr.2022.114396 35798129

[132] Gu C Geng X Wu Y Dai Y Zeng J Wang Z . Engineered macrophage membrane-coated nanoparticles with enhanced CCR2 expression promote spinal cord injury repair by suppressing neuroinflammation and neuronal death. Small. (2024) 20:e2305659. doi: 10.1002/smll.202305659 37884477

[133] Yan M Zhong Y Ni S Zhang S Jiang Y Zhu L . CCR2-engineered macrophage membrane-coated metal-polyphenol nanozyme to enhance antioxidation activity for inhibiting the atherosclerotic progression. Adv Healthc Mater. (2026) 15:e02151. doi: 10.1002/adhm.202502151 40974123

[134] Wang W Gao Y Zhang M Li Y Tang BZ . Neutrophil-like biomimic AIE nanoparticles with high-efficiency inflammatory cytokine targeting enable precise photothermal therapy and alleviation of inflammation. ACS Nano. (2023) 17:7394–405. doi: 10.1021/acsnano.2c11762 37009988

[135] Su T Huang K Ma H Liang H Dinh PU Chen J . Platelet-inspired nanocells for targeted heart repair after ischemia/reperfusion injury. Adv Funct Mater. (2019) 29:1803567. doi: 10.1002/adfm.201803567 32256277 PMC7111457

[136] Wang S Duan Y Zhang Q Komarla A Gong H Gao W . Drug targeting via platelet membrane-coated nanoparticles. Small Struct. (2020) 1:2000018. doi: 10.1002/sstr.202000018 33817693 PMC8011559

[137] Beetler DJ Bruno KA Watkins MM Xu V Chekuri I Giresi P . Reconstituted extracellular vesicles from human platelets decrease viral myocarditis in mice. Small. (2023) 19:e2303317. doi: 10.1002/smll.202303317 37612820 PMC10840864

[138] Dehaini D Wei X Fang RH Masson S Angsantikul P Luk BT . Erythrocyte-platelet hybrid membrane coating for enhanced nanoparticle functionalization. Adv Mater. (2017) 29:1606209. doi: 10.1002/adma.201606209 28199033 PMC5469720

[139] Kim YA Lee MH Sohn HS Kim HY . From circulation to regeneration: Blood cell membrane-coated nanoparticles as drug delivery platform for immune-regenerative therapy. Pharmaceutics. (2026) 18:66. doi: 10.3390/pharmaceutics18010066 41599174 PMC12844747

[140] Gao C Huang Q Liu C Kwong CHT Yue L Wan JB . Treatment of atherosclerosis by macrophage-biomimetic nanoparticles via targeted pharmacotherapy and sequestration of proinflammatory cytokines. Nat Commun. (2020) 11:2622. doi: 10.1038/s41467-020-16439-7 32457361 PMC7251120

[141] Rao L Xia S Xu W Tian R Yu G Gu C . Decoy nanoparticles protect against COVID-19 by concurrently adsorbing viruses and inflammatory cytokines. Proc Natl Acad Sci USA. (2020) 117:27141–7. doi: 10.1073/pnas.2014352117 33024017 PMC7959535

[142] Zhang Q Hu C Feng J Long H Wang Y Wang P . Anti-inflammatory mechanisms of neutrophil membrane-coated nanoparticles without drug loading. J Control Release. (2024) 369:12–24. doi: 10.1016/j.jconrel.2024.03.030 38508526

[143] Xiong Y Zhang Z Liu S Shen L Zheng L Ding L . T lymphocyte-macrophage hybrid membrane-coated biomimetic nanoparticles alleviate myocarditis via suppressing pyroptosis by targeting gene silencing. Int J Nanomedicine. (2024) 19:12817–33. doi: 10.21203/rs.3.rs-4206914/v1 39629104 PMC11614587

[144] Liu Z Yan J Zhou Y Yin M Zhou R Shen J . Biomimetic immunoregulators for the multi-target inflammation interruption in autoimmune diseases. ACS Nano. (2025) 19:27294–309. doi: 10.1021/acsnano.5c04655 40696743

[145] Han D Wang F Qiao Z Wang B Zhang Y Jiang Q . Neutrophil membrane-camouflaged nanoparticles alleviate inflammation and promote angiogenesis in ischemic myocardial injury. Bioact Mater. (2023) 23:369–82. doi: 10.1016/j.bioactmat.2022.11.016 36474655 PMC9706603

[146] Li Y Chen W Koo S Liu H Saiding Q Xie A . Innate immunity-modulating nanobiomaterials for controlling inflammation resolution. Matter. (2024) 7:3811–44. doi: 10.1016/j.matt.2024.09.016 40123651 PMC11925551

[147] Ala M . The beneficial effects of mesenchymal stem cells and their exosomes on myocardial infarction and critical considerations for enhancing their efficacy. Ageing Res Rev. (2023) 89:101980. doi: 10.1016/j.arr.2023.101980 37302757

[148] Golpanian S Wolf A Hatzistergos KE Hare JM . Rebuilding the damaged heart: Mesenchymal stem cells, cell-based therapy, and engineered heart tissue. Physiol Rev. (2016) 96:1127–68. doi: 10.1152/physrev.00019.2015 27335447 PMC6345247

[149] Sid-Otmane C Perrault LP Ly HQ . Mesenchymal stem cell mediates cardiac repair through autocrine, paracrine and endocrine axes. J Transl Med. (2020) 18:336. doi: 10.1186/s12967-020-02504-8 32873307 PMC7466793

[150] Konala VB Mamidi MK Bhonde R Das AK Pochampally R Pal R . The current landscape of the mesenchymal stromal cell secretome: A new paradigm for cell-free regeneration. Cytotherapy. (2016) 18:13–24. doi: 10.1016/j.jcyt.2015.10.008 26631828 PMC4924535

[151] Lotfy A AboQuella NM Wang H . Mesenchymal stromal/stem cell (MSC)-derived exosomes in clinical trials. Stem Cell Res Ther. (2023) 14:66. doi: 10.1186/s13287-023-03287-7 37024925 PMC10079493

[152] Nikfarjam S Rezaie J Zolbanin NM Jafari R . Mesenchymal stem cell derived-exosomes: a modern approach in translational medicine. J Transl Med. (2020) 18:449. doi: 10.1186/s12967-020-02622-3 33246476 PMC7691969

[153] Luther KM Haar L McGuinness M Wang Y Lynch TL IV Phan A . Exosomal miR-21a-5p mediates cardioprotection by mesenchymal stem cells. J Mol Cell Cardiol. (2018) 119:125–37. doi: 10.1016/j.yjmcc.2018.04.012 29698635

[154] Wei Z Chen Z Zhao Y Fan F Xiong W Song S . Mononuclear phagocyte system blockade using extracellular vesicles modified with CD47 on membrane surface for myocardial infarction reperfusion injury treatment. Biomaterials. (2021) 275:121000. doi: 10.1016/j.biomaterials.2021.121000 34218049

[155] Yan Q Song C Liu H Li Y Ma J Zhao Y . Adipose-derived stem cell exosomes loaded with icariin attenuated M1 polarization of macrophages via inhibiting the TLR4/Myd88/NF-κB signaling pathway. Int Immunopharmacol. (2024) 137:112448. doi: 10.1016/j.intimp.2024.112448 38870883

[156] Arabpour M Saghazadeh A Rezaei N . Anti-inflammatory and M2 macrophage polarization-promoting effect of mesenchymal stem cell-derived exosomes. Int Immunopharmacol. (2021) 97:107823. doi: 10.1016/j.intimp.2021.107823 34102486

[157] Yin K Wang S Zhao RC . Exosomes from mesenchymal stem/stromal cells: a new therapeutic paradigm. biomark Res. (2019) 7:8. doi: 10.1186/s40364-019-0159-x 30992990 PMC6450000

[158] de Almeida Fuzeta M Bernardes N Oliveira FD Costa AC Fernandes-Platzgummer A Farinha JP . Scalable production of human mesenchymal stromal cell-derived extracellular vesicles under serum-/xeno-free conditions in a microcarrier-based bioreactor culture system. Front Cell Dev Biol. (2020) 8:553444. doi: 10.3389/fcell.2020.553444 33224943 PMC7669752

[159] Sun X Shan A Wei Z Xu B . Intravenous mesenchymal stem cell-derived exosomes ameliorate myocardial inflammation in the dilated cardiomyopathy. Biochem Biophys Res Commun. (2018) 503:2611–8. doi: 10.1016/j.bbrc.2018.08.012 30126637

[160] Li X Li X Wang L Hou Y Liu Y Mao J . Advancing traditional Chinese medicine research through network pharmacology: strategies for target identification, mechanism elucidation and innovative therapeutic applications. Am J Chin Med. (2025) 53:2021–42. doi: 10.1142/s0192415x25500752 40884806

[161] Zhou Y Wang L Sun L Tan R Wang Z Pei R . Progress in Chinese medicine monomers and their nanoformulations on myocardial ischemia/reperfusion injury. J Mater Chem B. (2025) 13:1159–79. doi: 10.1039/d4tb02091j 39670754

[162] Zhuang Z Wang ZH Deng LH Zheng Q Zheng GQ Wang Y . Astragaloside IV exerts cardioprotection in animal models of viral myocarditis: a preclinical systematic review and meta-analysis. Front Pharmacol. (2019) 10:1388. doi: 10.3389/fphar.2019.01388 31849654 PMC6892970

[163] Chen D Xue C Liang Z Wang X Shi Z Yao M . Cardioprotective effects of puerarin against myocardial ischemia-reperfusion injury: a preclinical systematic review and meta-analysis. Front Pharmacol. (2026) 17:1770407. doi: 10.3389/fphar.2026.1770407 41924138 PMC13036222

[164] Xu J Chen X Guan X Zhang H Liu Y Zhang M . Therapeutic frontiers in viral myocarditis: targeting inflammation, viruses, oxidative stress, and myocardial repair. Front Immunol. (2025) 16:1643502. doi: 10.3389/fimmu.2025.1643502 40895553 PMC12390813

[165] Zhang J Zhao C Zhang W Zhang M Li F Shang H . Danhong injection targets CaMKII through dihydrotanshinone I to alleviate cardiomyocyte death and inflammation in viral myocarditis. Sci China Life Sci. (2026) 69:814–31. doi: 10.1007/s11427-025-2939-1 40889047

[166] Li L Hou X Xu R Liu C Tu M . Research review on the pharmacological effects of astragaloside IV. Fundam Clin Pharmacol. (2017) 31:17–36. doi: 10.1111/fcp.12232 27567103

[167] Yao J Liu J He Y Liu L Xu Z Lin X . Systems pharmacology reveals the mechanism of astragaloside IV in improving immune activity on cyclophosphamide-induced immunosuppressed mice. J Ethnopharmacol. (2023) 313:116533. doi: 10.1016/j.jep.2023.116533 37100262

[168] Huang H Lai S Wan Q Qi W Liu J . Astragaloside IV protects cardiomyocytes from anoxia/reoxygenation injury by upregulating the expression of Hes1 protein. Can J Physiol Pharmacol. (2016) 94:542–53. doi: 10.1139/cjpp-2015-0457 27070866

[169] Hammad H Lambrecht BN . Wnt and Hippo pathways in regulatory T cells: a NOTCH above in asthma. Nat Immunol. (2020) 21:1313–4. doi: 10.1038/s41590-020-0797-z 32968284 PMC7610869

[170] Yin X Liu B Wei H Wu S Guo L Xu F . Activation of the Notch signaling pathway disturbs the CD4(+)/CD8(+), Th17/Treg balance in rats with experimental autoimmune uveitis. Inflammation Res. (2019) 68:761–74. doi: 10.1007/s00011-019-01260-w 31209505

[171] Mohamed NM Abdelhamid AM Aref M Abdelhafeez M Faris Alotabi H Mohammed Abdelrahman DS . Role of cytokines and Th17/Tregs imbalance in the pathogenesis of otitis media with effusion. Modulation of Notch1/Hes1/mTORC1/S6k1 signalling pathway underlies the protective effect of astaxanthin. Int Immunopharmacol. (2024) 128:111521. doi: 10.1016/j.intimp.2024.111521 38246005

[172] Duan X Zhang L Liu K Guo K You Y Jia H . Macrophage-derived SPP1 exacerbate myocardial injury by interacting with fibroblasts in viral myocarditis. Biol Direct. (2025) 20:30. doi: 10.1186/s13062-025-00621-2 40087693 PMC11907792

[173] Wu S Zhou M Zhou H Han L Liu H . Astragaloside IV- loaded biomimetic nanoparticles target IκBα to regulate neutrophil extracellular trap formation for sepsis therapy. J Nanobiotechnology. (2025) 23:155. doi: 10.1186/s12951-025-03260-x 40022068 PMC11869569

[174] Li X Zhang X Qi Y Jin W Wen Z Zhao Y . Conductive bioadhesive hydrogel with controlled astragaloside IV release for ferroptosis-mediated cardiac repair. J Control Release. (2025) 384:113874. doi: 10.1016/j.jconrel.2025.113874 40414503

[175] Yan HW Feng YD Tang N Cao FC Lei YF Cao W . Viral myocarditis: from molecular mechanisms to therapeutic prospects. Eur J Pharmacol. (2024) 982:176935. doi: 10.1016/j.ejphar.2024.176935 39182550

[176] Esfandiarei M McManus BM . Molecular biology and pathogenesis of viral myocarditis. Annu Rev Pathol. (2008) 3:127–55. doi: 10.1146/annurev.pathol.3.121806.151534 18039131

[177] Liu P Martino T Opavsky MA Penninger J . Viral myocarditis: balance between viral infection and immune response. Can J Cardiol. (1996) 12:935–43. 9191484

[178] Laggerbauer B Engelhardt S . MicroRNAs as therapeutic targets in cardiovascular disease. J Clin Invest. (2022) 132:e159179. doi: 10.1172/jci159179 35642640 PMC9151703

[179] Liu C Wu R Yang H Yao Y . Immune cell dynamics and their role in cardiac injury: mechanisms and therapeutic implications. BioMed Pharmacother. (2025) 192:118608. doi: 10.1016/j.biopha.2025.118608 41016152

[180] Sun P Zhang Z Gao F Yang C Mang G Fu S . Silicate-based therapy for inflammatory dilated cardiomyopathy by inhibiting the vicious cycle of immune inflammation via FOXO signaling. Sci Adv. (2025) 11:eadr7208. doi: 10.1126/sciadv.adr7208 40203118 PMC11980853

[181] Maisch B . Cardio-immunology of myocarditis: focus on immune mechanisms and treatment options. Front Cardiovasc Med. (2019) 6:48. doi: 10.3389/fcvm.2019.00048 31032264 PMC6473396

[182] Gross CC Schulte-Mecklenbeck A Steinberg OV Wirth T Lauks S Bittner S . Multiple sclerosis endophenotypes identified by high-dimensional blood signatures are associated with distinct disease trajectories. Sci Transl Med. (2024) 16:eade8560. doi: 10.1126/scitranslmed.ade8560 38536936

[183] Yang Y Zhang Y Wu J Liu Y Lei X . Decoding immune low-response states in sepsis: single-cell and 3D spatial transcriptomic insights into immunoparalysis. Front Immunol. (2025) 16:1696914. doi: 10.3389/fimmu.2025.1696914 41268561 PMC12626914

[184] Olejniczak M Schwartz M Webber E Shaffer A Perry TE . Viral myocarditis-incidence, diagnosis and management. J Cardiothorac Vasc Anesth. (2020) 34:1591–601. doi: 10.1053/j.jvca.2019.12.052 32127272

[185] Heymans S Eriksson U Lehtonen J Cooper LT Jr . The quest for new approaches in myocarditis and inflammatory cardiomyopathy. J Am Coll Cardiol. (2016) 68:2348–65. doi: 10.1016/j.jacc.2016.09.937 27884253

[186] Maisch B Herzum M Hufnagel G Schonian U . Immunosuppressive and immunomodulatory treatment for myocarditis. Curr Opin Cardiol. (1996) 11:310–24. doi: 10.1097/00001573-199605000-00012 8835874

[187] Kyaw T Drummond G Bobik A Peter K . Myocarditis: causes, mechanisms, and evolving therapies. Expert Opin Ther Targets. (2023) 27:225–38. doi: 10.1080/14728222.2023.2193330 36946552

[188] Mangan MSJ Olhava EJ Roush WR Seidel HM Glick GD Latz E . Targeting the NLRP3 inflammasome in inflammatory diseases. Nat Rev Drug Discov. (2018) 17:588–606. doi: 10.1038/nrd.2018.97 30026524

[189] Del Buono MG Bonaventura A Vecchié A Moroni F Golino M Bressi E . Pathogenic pathways and therapeutic targets of inflammation in heart diseases: a focus on interleukin-1. Eur J Clin Invest. (2024) 54:e14110. doi: 10.1111/eci.14110 37837616

[190] Carai P González LF Van Bruggen S Spalart V De Giorgio D Geuens N . Neutrophil inhibition improves acute inflammation in a murine model of viral myocarditis. Cardiovasc Res. (2023) 118:3331–45. doi: 10.1093/cvr/cvac052 35426438 PMC9847559

[191] Lee M Kim MC Lee JY . Nanomaterial-based electrically conductive hydrogels for cardiac tissue repair. Int J Nanomedicine. (2022) 17:6181–200. doi: 10.2147/ijn.s386763 36531116 PMC9748845

[192] Webb C Forbes N Roces CB Anderluzzi G Lou G Abraham S . Using microfluidics for scalable manufacturing of nanomedicines from bench to GMP: a case study using protein-loaded liposomes. Int J Pharm. (2020) 582:119266. doi: 10.1016/j.ijpharm.2020.119266 32251694

[193] Dri DA Rinaldi F Carafa M Marianecci C . Nanomedicines and nanocarriers in clinical trials: surfing through regulatory requirements and physico-chemical critical quality attributes. Drug Delivery Transl Res. (2023) 13:757–69. doi: 10.1007/s13346-022-01262-y 36450964 PMC9713170

[194] Moura D Rohringer S Ferreira HP Pereira AT Barrias CC Magalhães FD . Long-term *in vivo* degradation and biocompatibility of degradable pHEMA hydrogels containing graphene oxide. Acta Biomater. (2024) 173:351–64. doi: 10.1016/j.actbio.2023.11.012 37984630

[195] Cherbi M Gautier P Sacher F Martins R Knecht S Maury P . Ventricular arrhythmias following left ventricular assist device implantation: a systematic review and meta-analysis of incidence and clinical impact. Europace. (2025) 27:euaf129. doi: 10.1093/europace/euaf129 40561522 PMC12246684

[196] Harding D Chong MHA Lahoti N Bigogno CM Prema R Mohiddin SA . Dilated cardiomyopathy and chronic cardiac inflammation: pathogenesis, diagnosis and therapy. J Intern Med. (2023) 293:23–47. doi: 10.1111/joim.13556 36030368

[197] Martens P Cooper LT Tang WHW . Diagnostic approach for suspected acute myocarditis: considerations for standardization and broadening clinical spectrum. J Am Heart Assoc. (2023) 12:e031454. doi: 10.1161/jaha.123.031454 37589159 PMC10547314

[198] Han K Mao M Fu L Zhang Y Kang Y Li D . Multimaterial printing of serpentine microarchitectures with synergistic mechanical/piezoelectric stimulation for enhanced cardiac-specific functional regeneration. Small. (2024) 20:e2401561. doi: 10.1002/smll.202401561 38899348

